# Entrainment and synchronization in networks of Rayleigh–van der Pol oscillators with diffusive and Haken–Kelso–Bunz couplings

**DOI:** 10.1007/s00422-016-0685-7

**Published:** 2016-04-23

**Authors:** Francesco Alderisio, Benoît G. Bardy, Mario di Bernardo

**Affiliations:** Department of Engineering Mathematics, Merchant Venturers’ Building, University of Bristol, Woodland Road, Clifton, Bristol, BS8 1UB UK; EuroMov, Montpellier University, 700 Avenue du Pic Saint-Loup, 34090 Montpellier, France; Institut Universitaire de France, 1 rue Descartes, 75231 Paris Cedex 05, France; Department of Electrical Engineering and Information Technology, University of Naples Federico II, Via Claudio 21, 80125 Naples, Italy

**Keywords:** Group synchronization, Human coordination, Heterogeneous networks, Nonlinear oscillators, HKB coupling, Entrainment

## Abstract

We analyze a network of non-identical Rayleigh–van der Pol (RvdP) oscillators interconnected through either diffusive or nonlinear coupling functions. The work presented here extends existing results on the case of two nonlinearly coupled RvdP oscillators to the problem of considering a network of three or more of them. Specifically, we study synchronization and entrainment in networks of heterogeneous RvdP oscillators and contrast the effects of diffusive linear coupling strategies with the nonlinear Haken–Kelso–Bunz coupling, originally introduced to study human bimanual experiments. We show how convergence of the error among the nodes’ trajectories toward a bounded region is possible with both linear and nonlinear coupling functions. Under the assumption that the network is connected, simple, and undirected, analytical results are obtained to prove boundedness of the error when the oscillators are coupled diffusively. All results are illustrated by way of numerical examples and compared with the experimental findings available in the literature on synchronization of people rocking chairs, confirming the effectiveness of the model we propose to capture some of the features of human group synchronization observed experimentally in the previous literature.

## Introduction

Interpersonal coordination and synchronization between the motion of two individuals have been extensively studied over the past few decades (Schmidt and Turvey 1994; Varlet et al. 2011). Synergetic movements of two or more people mirroring each other frequently occur in many activities such as handling objects, manipulating a common workpiece, dancing, choir singing, and movement therapy (Himberg and Thompson 2009; Valdesolo et al. 2010; Mörtl et al. 2012; Lorenz et al. 2013; Repp and Su 2013). It is of great importance to reveal not only the effects of mirroring movements among people on human physiological and mental functions, but also to understand the link between intrapersonal and interpersonal coordination. In social psychology, it has been shown that people prefer to team up with others possessing similar morphological and behavioral features and that they tend to coordinate their movement unconsciously (Folkes 1982; Lakens and Stel 2011). Moreover, much evidence suggests that interpersonal motor coordination is strictly related to social attachment, meaning that synchronous activities between individuals, that occur even more when the kinematic features of their movements share similar patterns (Słowiński et al. 2016), may produce positive emotions (Wiltermuth and Heath 2009).

In order to explain the experimental observations of human interpersonal coordination, mathematical models are usually derived to capture the key features of the observed behavior. A classical example is the nonlinear RvdP oscillator with HKB coupling, which was introduced in Haken et al. (1985) to explain the transition from phase to antiphase synchronization in bimanual coordination experiments (for more details, see Kelso et al. 1987; Jirsa et al. 1998). Such model was shown to be able to capture many features of human coordination even beyond the bimanual synchronization experiments it was derived to explain. For example, RvdP oscillators were used in Zhai et al. (2014a, b, 2015a, b, c); Alderisio et al. 2016) to design virtual players in the context of the *mirror game* (Noy et al. 2011), presented as an important paradigmatic case of study to investigate the onset of social motor coordination between two players imitating each other’s hand movements. Furthermore, in Kelso et al. (2009) the authors take inspiration from the *dynamic clamp* of cellular and computational neuroscience in order to probe essential properties of human social coordination by reciprocally coupling human subjects to a computationally implemented model of themselves (RvdP oscillator with HKB coupling), referred to as virtual player. Such concept, namely the *human dynamic clamp*, was further investigated and developed in Dumas et al. (2014) in order to cover a broader repertoire of human behavior, including rhythmic and discrete movements, adaptation to changes of pacing, and behavioral skill learning as specified by the virtual player. Besides, RvdP oscillators were also used in Schmidt and Turvey (1994) in order to capture the rhythmic coordination between two individuals swinging handheld pendulums, in Varlet et al. (2011) in order to model spontaneous interpersonal postural coordination between two human people and account for the competition between the coupling to a visual target to track and the coupling to the partner, in Richardson et al. (2007) in order to qualitatively explain interpersonal movement synchronization between two human beings involved in rhythmic paradigms, and in Amazeen et al. (1995) in order to account for the frequency detuning of the phase entrainment dynamics of two people involved in interlimb coordination.

While coordination of two human players has been studied in numerous previous investigations, the case of multiple human players has been seldom studied in the existing literature, due to a combination of practical problems in running the experiments and lack of a formal method able not only to model the considered scenario but also to quantify and characterize the synchronization level of the ensemble. Multiplayer games involve a group of three or more people engaged in a communal coordination task. The variety of scenarios that can be considered is vast due to the countless activities the players might be involved in (limb movements, finger movements, head movements, walking in a crowd, or more in general music and sport activities), the many ways in which the participants can interact and communicate with each other and the different ways all the players can be physically located with respect to each other while performing the specified task.

Some of the existing works on coordination of multiple human players include studies on choir singers during a concert (Himberg and Thompson 2009), rhythmic activities as, for example, “the cup game” and marching tasks (Iqbal and Riek 2015), rocking chairs (Frank and Richardson 2010; Richardson et al. 2012), and coordination of rowers’ movements during a race (Wing and Woodburn 1995). In these papers, the authors provide several experimental results in order to analyze the behavior of a group of people performing some coordinated activities, but a rigorous mathematical model capable of capturing the observed results and explaining the features of the movement coordination among them is still missing. In particular, in Frank and Richardson (2010) the authors study both unintentional and intentional coordination by asking the players to try and synchronize the oscillations of the rocking chairs they are sitting on with their eyes shut or open. Synchronization is observed to spontaneously emerge when players observe each other’s movements. Another study in which multiplayer activities are analyzed but a mathematical model is missing is carried out in Yokoyama and Yamamoto (2011) where the theory of symmetric Hopf bifurcations in coupled oscillators is used to investigate the synchronized patterns of three people during sport activities.

Further results about multiplayer activities deal with spontaneous group synchronization of arm movements and respiratory rhythms. For example, in Codrons et al. (2014) the authors test whether pre-assigned arm movements performed in a group setting spontaneously synchronize and whether synchronization extends to heart and respiratory rhythms. In their study, no explicit directions are given on whether or how the arm swingings are to be synchronized among participants, and experiments are repeated with and without external cues. Interestingly, when an external auditory rhythm is present, both motor and respiratory synchronization is found to be enhanced among the group. Also, the overall coordination level is observed to increase when compared to that detected when the same experiments are again carried out in the absence of the external cue.

The main objective of this paper is to propose and analyze a model able to account for movement synchronization in multiplayer scenarios and explain some of the features observed experimentally in the existing literature. Specifically, we consider networks of heterogeneous nonlinear RvdP oscillators as a good model of multiplayer coordination and, as already done in Mörtl et al. (2012) for the case of two agents only, we regard it as a synchronization problem. Each equation is used to model the movement of a different player and is therefore characterized by a different set of parameters to account for human-to-human variability. The effects of different interaction models, linear and nonlinear, are investigated to understand under what conditions synchronization is observed to emerge. Our analysis suggests that bounded synchronization is indeed a common emergent property in these networks whose occurrence can also be accounted for analytically in a number of different scenarios. Also, as expected from existing theoretical results, we find that the structure of the interactions among players has an effect on the coordination level detected in the network.

Furthermore, the effects of adding an external sinusoidal signal are studied in order to understand whether synchronization can be improved by means of an entrainment signal (Russo et al. 2010). Our analysis suggests that the coordination level of the ensemble can indeed increase when the oscillation frequency of the external signal is similar to the natural angular velocity of the agents in the network. However, in all the other cases, the external signal acts as a disturbance and leads to a decrease in the coordination among the agents.

We wish to emphasize that the study reported in this paper will form the basis of future experimental investigations which are currently being planned.

The rest of the paper is organized as follows. In Sect. 2, some notation that will be used in later sections is introduced. In Sect. 3, the equation that describes the network is presented, in terms of both internal dynamics of each agent and coupling function thanks to which they can interact with each other. In Sect. 4, some metrics are introduced to characterize the quality and the level of coordination in human groups. In Sect. 5, a testbed scenario of multiplayer coordination in networks of human people is presented, while in Sect. 5.1, the key synchronization features experimentally observed are reproduced by considering a network of heterogeneous RvdP oscillators, and the effects of three different coupling strategies thanks to which they are interconnected are explored. In Sect. 6, the effects of adding an external entrainment signal are analyzed with respect to the overall coordination level of the network. In Sect. 7, bounded synchronization of the network when its nodes are connected through a linear diffusive coupling function is analytically proven to be achieved, and some numerical examples are provided in order to both illustrate the effectiveness of our analysis and to show that bounded synchronization can be achieved also when considering different couplings. Finally, in Sect. 8, a summary of our results and some possible future developments are presented.

## Preliminaries and background

We denote with $\otimes$ the Kronecker product between two matrices. The operator $\lambda _k \left( \cdot \right)$ defined over a matrix indicates the *k*th eigenvalue of the matrix itself, and $\lambda _M \left( \cdot \right)$ indicates its maximum eigenvalue when the matrix is real and symmetric and as a consequence all the eigenvalue are real as well.

A *graph* is a tuple ${\mathcal {G}} = \{ {\mathcal {V}}, {\mathcal {E}} \}$ defined by a set of nodes ${\mathcal {V}} = \{ 1,\ldots ,N \}$ and a set of edges ${\mathcal {E}} \subseteq {\mathcal {V}} \times {\mathcal {V}}$. A graph is said to be *undirected* if $(i,j) \in {\mathcal {E}} \iff (j,i) \in {\mathcal {E}}$. In an undirected graph, two nodes *i* and *j* are said to be *neighbors* if $(i,j) \in {\mathcal {E}}$. The matrix $A=\{a_{ij} \} \in {\mathbb {R}}^{N \times N}$, where

$$\begin{aligned} a_{ij}{\left\{ \begin{array}{ll} >0, &{} \text{ if } (i,j) \text{ are } \text{ neighbors } \\ =0, &{} \text{ otherwise } \end{array}\right. } \end{aligned}$$

is called *adjacency matrix*, and $a_{ij} \ge 0$ is called strength of the interaction between the pair (*i*, *j*). In particular, a graph is said to be *unweighted* if the interaction between two neighbors is equal to 1. A *path* between nodes *h* and *k* is a sequence of nodes, with *h* and *k* as endpoints, such that every two consecutive nodes are neighbors. A graph is said to be *simple* if $a_{ii} = 0 \ \forall i \in {\mathcal {V}}$, and it is said to be *connected* if there exists a path between any two of its nodes. The matrix $L = \{l_{ij} \} \in {\mathbb {R}}^{N \times N}$ defined as

1 $$\begin{aligned} l_{ij} : = {\left\{ \begin{array}{ll} \sum _{k=1}^{N} a_{ik}, &{} \text{ if } i=j \\ -a_{ij}, &{} \text{ if } i \ne j \end{array}\right. } \end{aligned}$$

is called *Laplacian matrix* of the graph (or simply *Laplacian*). The Laplacian of any simple undirected graph is symmetric with zero row sum and is a positive semidefinite matrix with as many null eigenvalues as there are components in the graph. In particular, a connected graph has only one null eigenvalue.

Throughout the paper, we will consider a connected simple undirected network of *N* agents assuming that any two players interact symmetrically with one another.

Before analyzing a multiplayer scenario, it is worth considering the simpler case of only two human players interacting with each other. The system that can be used to model the interaction between them is described in terms of two coupled RvdP oscillators and can be given as follows (Richardson et al. 2007; Fuchs and Jirsa 2008):

2 $$\begin{aligned} {\left\{ \begin{array}{ll} \ddot{x_1}+\left( \alpha x_1^2 + \beta \dot{x}_1^2 -\gamma \right) \dot{x}_1 + \omega _1^2 x_1 = I(x_1,x_2) \\ \ddot{x_2}+\left( \alpha x_2^2 + \beta \dot{x}_2^2 -\gamma \right) \dot{x}_2 + \omega _2^2 x_2 = I(x_2,x_1) \end{array}\right. } \end{aligned}$$

where $x_i \in {\mathbb {R}}$ denotes the position of the *i*th player, with $i=1,2$. The right-hand side of both equations represents the coupling term between the two players: in particular, using the model proposed by Haken, Kelso and Bunz in Haken et al. (1985), it is given by

3 $$\begin{aligned} I(w,z) := [ a+b\left( w-z \right) ^2 ] \left( \dot{w}-\dot{z} \right) \end{aligned}$$

The term $\left( \alpha x_i^2 + \beta \dot{x}_i^2 -\gamma \right) \dot{x}_i$ represents the nonlinear damping of the oscillatory movement of player *i*. Specifically, the sign of $\gamma$ determines whether, in the absence of coupling, the oscillation is persistent ($\gamma >0$) or vanishes (vice versa) as time goes by: it is trivial to verify this by studying the stability of the origin and checking the sign of the eigenvalues of the Jacobian of the system (Avitabile et al. 2015). Moreover, $\alpha$ and $\beta$ determine the amplitude of such oscillation, while $\omega _i$ is related to its frequency. It has been proven that this model of two nonlinearly coupled oscillators accounts for the features observed during experimental data in bimanual experiments (see Haken et al. (1985) for further details).

## Human-to-human coordination as a synchronization problem

We have pointed out that the dynamics of two coupled RvdP oscillators has been used to describe different kinds of interpersonal coordination tasks between two people, including bimanual coordination experiments, mirror game, social postural coordination, and rocking chairs. According to the particular scenario considered, the state vector of each oscillator is used to represent position and velocity of the particular body part of interest of either of the players (finger, hand, head, and so forth). Following the same approach, we can consider a scenario in which more than two human beings are performing a multiplayer coordination task involving some oscillatory motion, as, for example, arm or hand rhythmic movements, rocking chairs, and head tracking of a visual target. In these cases, the state vector of each node represents position and velocity of the particular body part of interest of each player. Therefore, the dynamics of each player when moving in isolation will be described by the following nonlinear system:

4 $$\begin{aligned} f_i(t,x_i)= \begin{bmatrix} x_{i_2} \\ - (\alpha _i x_{i_1}^2+\beta _i x_{i_2}^2-\gamma _i)x_{i_2} - \omega _i^2 x_{i_1} \end{bmatrix} \end{aligned}$$

where $x_i=[x_{i_1} \ x_{i_2}]^T \in {\mathbb {R}}^2$ is the state vector, with $x_{i_1}, x_{i_2}$ representing position and velocity of the *i*th human player, respectively.

To model the interaction between different players, we assume that the dynamics of each of them is affected by some coupling function $u_i$ which depends on the difference between the state of the *i*th player and that of his/her neighbors. In what follows, we will explore the effects of three possible selections for such a function. We are interested in analyzing which one leads to synchronization features which are the closest to those observed in previous experimental work about human ensembles involved in a joint coordination task, e.g. (Richardson et al. 2012).

1. *Full state coupling*. With this kind of coupling, we assume that players adjust both their velocities and accelerations proportionally to the average mismatch between their own position and velocity and those of their neighbors. Mathematically, we have:
  5 $$\begin{aligned} u_i = -\frac{c}{{\mathcal {N}}_i} \sum _{j=1}^{N} a_{ij} \left( x_i-x_j \right) \end{aligned}$$
  In particular, ${\mathcal {N}}_i>0$ is the number of neighbors of node *i*, while $c>0$ is the coupling strength among the agents.
2. *Partial state coupling*. Next, we explore the case where players only adjust their accelerations according to the position and velocity mismatches from their neighbors:
  6 $$\begin{aligned} u_i = - \begin{bmatrix} 0 \\ \sum _{j=1}^{N} \frac{a_{ij}}{{\mathcal {N}}_i} \left[ c_1 \left( x_{i_1}-x_{j_1} \right) + c_2 \left( x_{i_2}-x_{j_2} \right) \right] \end{bmatrix}\nonumber \\ \end{aligned}$$
  In particular, ${\mathcal {N}}_i>0$ is the number of neighbors of node *i*, while $c_1,c_2>0$ represent the position and the velocity coupling strengths, respectively.
3. *HKB coupling*. Finally, we consider an interaction model which is the direct extension to multiplayer coordination problems of the interaction function used to describe the bimanual experiments (Haken et al. 1985; Fuchs et al. 1996). Specifically, we choose the following nonlinear function:
  7 $$\begin{aligned} u_i = \begin{bmatrix} 0 \\ \frac{c}{{\mathcal {N}}_i} \sum _{j=1}^{N} a_{ij} [a+b(x_{i_1}-x_{j_1})^2](x_{i_2}-x_{j_2}) \end{bmatrix} \end{aligned}$$
  Once again, ${\mathcal {N}}_i>0$ is the number of neighbors of node *i*, while $c>0$ represents the coupling strength among the agents.

The resulting network model describing the interaction of a group of *N* players can then be written as

8 $$\begin{aligned} \dot{x}_i(t) \!= \!\begin{bmatrix} x_{i_2} \\ \!-\! (\alpha _i x_{i_1}^2+\beta _i x_{i_2}^2-\gamma _i)x_{i_2} \!-\! \omega _i^2 x_{i_1} \end{bmatrix} + u_i(t) \ \in {\mathbb {R}}^2\nonumber \\ \end{aligned}$$

where the coupling function $u_i$ can be chosen as one of those listed above. We now explore under what conditions coordination, and hence synchronization, emerges for each of the three scenarios of interest.

We wish to emphasize that, since the node parameters are heterogeneous, complete synchronization as defined in Li et al. (2010) cannot be achieved. We will consider instead the case where bounded synchronization, as defined in Hill and Zhao (2008) and below, emerges. Namely, we define the average trajectory as

9 $$\begin{aligned} \bar{x}(t) : = \frac{1}{N} \sum _{j=1}^{N} x_j(t) \end{aligned}$$

and its distance from the state of each node *i* as

10 $$\begin{aligned} e_i(t) : = x_i(t)-\bar{x}(t) \quad \forall t \ge 0, i=1,\ldots ,N \end{aligned}$$

We also define the parameters vector for each node *i* as $\vartheta _i := [\alpha _i \ \beta _i \ \gamma _i \ \omega _i]^T \in {\mathbb {R}}^4$, and we introduce the stack vectors $x(t) : = [x_1(t)^T \ x_2(t)^T \ \ldots \ x_N(t)^T]^T \in {\mathbb {R}}^{2N}$ and $e(t) : = [e_1(t)^T \ e_2(t)^T \ \ldots \ e_N(t)^T]^T \in {\mathbb {R}}^{2N}$ and the error norm $\eta (t) : = ||e(t)|| \in {\mathbb {R}}, \forall t \ge 0$, where $|| \cdot ||$ indicates the Euclidean norm.

### **Definition 1**

We say that a network of non-identical RvdP oscillators achieves *bounded synchronization* if and only if there exists some time instant $\hat{t}$ such that

11 $$\begin{aligned} \eta (t) \le \epsilon \quad \forall t>\hat{t} \end{aligned}$$

for any initial condition $x_{i,0}$ and parameter vector $\vartheta _i$ of the nodes in the network.

### **Definition 2**

If a network of non-identical RvdP oscillators achieves bounded synchronization, we define the *relative synchronization error bound*$\chi$ as the upper bound of the ratio between the error norm $\eta (t)$ when the oscillators are coupled, and its maximum value $\tilde{\eta }$ when they are uncoupled, that is

12 $$\begin{aligned} \frac{\eta (t)}{\tilde{\eta }} \le \chi \quad \forall t >\hat{t} \end{aligned}$$

According to Definition 1, we will say that the network has achieved bounded synchronization if the error norm reaches and remains into a bounded region for all $t>\hat{t}$. We will then use the relative synchronization error bound $\chi$, as described in Definition 2, in order to evaluate the improvement in synchronization among the oscillators when coupling through the network is present.

Assuming that when the nodes are coupled $\eta (t) \le \tilde{\eta }$, we have that $\chi \in [0,1]$. In particular, $\chi =0$ represents the ideal case of complete synchronization, while $\chi =1$ represents the worst-case scenario. The relative synchronization error bound can be lowered, in general, by increasing the strength of the coupling function (e.g., see Theorem 2 and Sect. 7.3).

## Coordination metrics

In order to quantify and analyze the coordination level in a network of more than two agents, we use the metrics introduced in Richardson et al. (2012) to characterize the quality of synchronization in human groups.

Let $x_k(t) \in {\mathbb {R}} \ \forall t \in [0,T]$ be the continuous time series representing the motion of each agent, with $k \in [1,N]$, where *N* is the number of individuals and *T* is the duration of the experiment. Let $x_k(t_i) \in {\mathbb {R}}$, with $k \in [1,N]$ and $i \in [1,N_T]$, be the respective discrete time series of the *k*th agent, obtained after sampling $x_k(t)$, where $N_T$ is the number of time steps and ${\Delta } T := \frac{T}{N_T}$ is the sampling period. Let $\theta _k(t) \in [-\pi ,\pi ]$ be the phase of the *k*th agent, which can be estimated by making use of the Hilbert transform of the signal $x_k(t)$ (Kralemann et al. 2008). We define the *cluster phase* or *Kuramoto order parameter*, both in its complex form $q'(t) \in {\mathbb {C}}$ and in its real form $q(t) \in [-\pi ,\pi ]$ as

13 $$\begin{aligned} q'(t):= & {} \frac{1}{N} \sum _{k=1}^{N} e^{ j \theta _k(t) } \end{aligned}$$

14 $$\begin{aligned} q(t):= & {} \mathrm{atan2} \left( \mathfrak {I}(q'(t)),\mathfrak {R}(q'(t)) \right) \end{aligned}$$

which can be regarded as the average phase of the group at time *t*.

Let $\phi _k(t) := \theta _k(t) - q(t)$ be the relative phase between the *k*th participant and the group phase at time *t*. We can define the relative phase between the *k*th participant and the group averaged over the time interval $[t_1,t_{N_T}]$, both in its complex form $\bar{\phi }'_k \in {\mathbb {C}}$ and in its real form $\bar{\phi }_k \in [-\pi ,\pi ]$ as

15 $$\begin{aligned} \bar{\phi }'_k:= & {} \frac{1}{T} \int _{0}^{T} e^{ j \phi _k(t) } \ {\text {d}}t \simeq \frac{1}{N_T} \sum _{i=1}^{N_T} e^{ j \phi _k(t_i) } \end{aligned}$$

16 $$\begin{aligned} \bar{\phi }_k:= & {} \mathrm{atan2} \left( \mathfrak {I}(\bar{\phi }'_k), \mathfrak {R}(\bar{\phi }'_k) \right) \end{aligned}$$

In order to quantify the degree of synchronization for the *k*th agent within the group, we define the following order parameter

17 $$\begin{aligned} \rho _k := |\bar{\phi }'_k| \quad \in [0,1] \end{aligned}$$

which simply gives information on how much the *k*th agent is synchronized with the average trend of the group. The closer $\rho _k$ is to 1, the better the synchronization of the *k*th agent itself.

In order to quantify the coordination level of the entire group at time *t*, following Richardson et al. (2012), we define the following parameter

18 $$\begin{aligned} \rho _{g}(t) := \frac{1}{N} \left| \sum _{k=1}^{N} e^{j {\Delta } \phi _k(t)} \right| \quad \in [0,1] \end{aligned}$$

which simply represents the group synchronization, with ${\Delta } \phi _k(t):=\phi _k(t)- \bar{\phi }_k$. The closer $\rho _{g}(t)$ is to 1, the better the coordination level of the group at time *t*. Its value can be averaged over the whole time interval [0, *T*] in order to have an estimate of the mean coordination level of the group during the total duration of the performance:

19 $$\begin{aligned} \rho _g := \frac{1}{T} \int _{0}^{T} \rho _{g}(t) \ {\text {d}}t \simeq \frac{1}{N_T} \sum _{i=1}^{N_T} \rho _{g}(t_i) \quad \in [0,1] \end{aligned}$$

Besides, if we denote with $\phi _{d_{k,h}}(t):=\theta _k(t)-\theta _{h}(t)$ the relative phase between two participants in the group at time *t*, it is possible to estimate their dyadic synchronization, that is the coordination level between participants *k* and *h* over the total duration *T* of the trial (Richardson et al. 2012):

20 $$\begin{aligned} \rho _{d_{k,h}}:= & {} \left| \frac{1}{T} \int _{0}^{T} e^{ j \phi _{d_{k,h}}(t) } \ {\text {d}}t \right| \nonumber \\\simeq & {} \left| \frac{1}{N_T} \sum _{i=1}^{N_T} e^{ j \phi _{d_{k,h}}(t_i) } \right| \quad \in [0,1] \end{aligned}$$

Note that high dyadic coordination levels can coexist with low group synchronization values.

Also note that, in general, the tighter bounded synchronization is (i.e., the smaller $\epsilon$ in Eq. 11 or equivalently $\chi$ in Eq. 12), the higher is the group synchronization index defined in Eq. 18. Intuitively, the closer the trajectories $x_k(t)$ of all the nodes are, the closer are their phases $\theta _k(t)$, and so are their differences with the phase of the group $\phi _k(t)$ and their respective values averaged over time $\bar{\phi }_k$. A numerical example of such relationship is reported in Sect. 7.3.

### *Remark 1*

For strong coupling, the HKB model entails bistable coordination as an important feature; hence, it is possible that the network converges toward regimes where some of the oscillators evolve in anti-phase. According to our definitions, these scenarios correspond to unwanted situations. Indeed, we focus on the case where all the oscillators exhibit relatively small phase differences corresponding to large values of the group synchronization index (typically over 90 %).

## Testbed example

As a testbed scenario, we consider the synchronization of rocking chairs motion studied in Richardson et al. (2012). In particular, participants sit on six identical wooden rocking chairs disposed as a circle and are supposed to rock them in two different conditions:

1. *Eyes closed*: participants are required to rock at their own preferred frequency while keeping their eyes closed;
2. *Eyes open*: participants are required to rock at their own preferred frequency while trying to synchronize their rocking chair movements as a group.

In the eyes closed condition, the participants are not visually coupled, meaning that the oscillation frequency of each of them is not influenced by the motion of the others, while in the eyes open condition each player is asked to look at the middle of the circle in order to try and synchronize their motion with that of the others. The six participants first perform a trial while keeping their eyes closed and then perform two eyes open trials, namely T1 and T2, each lasting 3 min.

**Fig. 1:**
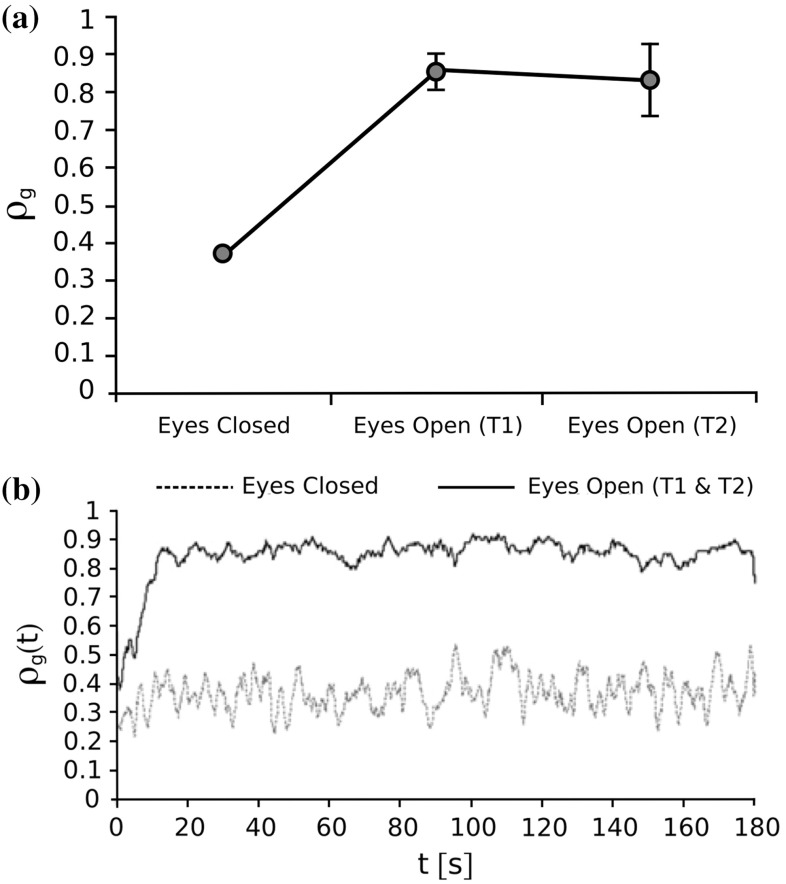
Group synchronization in the rocking chairs experiments, adapted from Richardson et al. (2012)—T1 and T2 refer to two different trials of the eyes open condition. **a** Mean value (circle) and standard deviation (error bar), **b** typical trend

In Fig. 1, the typical trend of the group synchronization index $\rho _g(t)$ and its mean value and standard deviation are depicted for each of the three aforementioned trials. In particular, in Fig. 1a it is possible to appreciate that the mean value $\rho _g$ of the group synchronization index is around 0.4 in the eyes closed condition, while it is around 0.85 in the eyes open condition. This means that, when the participants are not visually coupled, as expected synchronization does not emerge, while when the participants are visually coupled and explicitly told to rock their own chair movements as a group, the coordination level significantly increases. In Fig. 1b, it is possible to appreciate that in the eyes closed condition the amplitude of the oscillations of the group synchronization is higher than that observed during the eyes open trials.

In Table 1, we show typical values of the degree of synchronization $\rho _k$ of the participants involved in the rocking chairs experiments, for both the eyes closed and the eyes open condition. It is possible to appreciate how, as expected, the value of $\rho _k$ is much higher for almost all the participants when they are visually coupled. Interestingly enough, agent 6 does not undergo an improvement of $\rho _6$ with respect to the eyes closed condition, meaning that such participant has more trouble synchronizing with the group compared to the others.

**Table 1:** Degree of synchronization of the participants in the rocking chairs experiments of Richardson et al. (2012)

Participant	EC ($\rho _g=0.36$)	EO ($\rho _g=0.80$)
1	0.36	0.95
2	0.34	0.92
3	0.30	0.95
4	0.35	0.88
5	0.34	0.67
6	0.40	0.37

*EC* eyes closed, *EO* eyes open

In Table 2, we show typical values of the dyadic synchronization $\rho _{d_{k,h}}$ between participants involved in the rocking chairs experiments for the eyes open condition. As expected, the lowest values are those corresponding to the participant that struggled the most to synchronize with the rest of the group, that is participant 6.

**Table 2:** Dyadic synchronization of the participants in the rocking chairs experiments of Richardson et al. (2012) for the eyes open condition ($\rho _g=0.80$)

Participants	2	3	4	5	6
1	0.87	0.86	0.81	0.63	0.19
2	–	0.85	0.78	0.59	0.21
3	–	–	0.82	0.61	0.21
4	–	–	–	0.50	0.18
5	–	–	–	–	0.14

### Modeling results

In this section, we uncover the synchronization features that the three different coupling functions introduced earlier lead to, with respect to the rocking chairs experiments introduced earlier as a testbed scenario (Richardson et al. 2012). We will explore whether and how the model of coupled RvdP oscillators we propose in this paper can reproduce the key features of the observed experimental results. In so doing, we will explore:

- the effects of choosing different coupling functions;
- how varying the coupling strength affects the coordination level of the agents.

We simulate a network of $N=6$ heterogeneous RvdP oscillators whose parameters and initial values are heuristically set as described in Table 3 and we set $T=200$ s. We suppose that the network is simple, connected, unweighted, and undirected and we assume that each node is connected to all the others (complete graph), which we believe well represents the topology implemented in the rocking chairs experiments of Richardson et al. (2012) for the eyes open condition.

**Table 3:** Numerical simulations—parameters and initial values for a network of $N=6$ heterogeneous RvdP oscillators

Node	$\alpha _i$	$\beta _i$	$\gamma _i$	$\omega _i$	$x_i(0)$
1	0.46	1.16	0.58	0.31	$[-1.4, +0.3]$
2	0.37	1.20	1.84	0.52	$[+1.0, +0.2]$
3	0.34	1.73	0.62	0.37	$[-1.8, -0.3]$
4	0.17	0.31	1.86	0.41	$[+0.2, -0.2]$
5	0.76	0.76	1.40	0.85	$[+1.5, +0.1]$
6	0.25	0.86	0.56	0.62	$[-0.8, -0.1]$

**Fig. 2:**
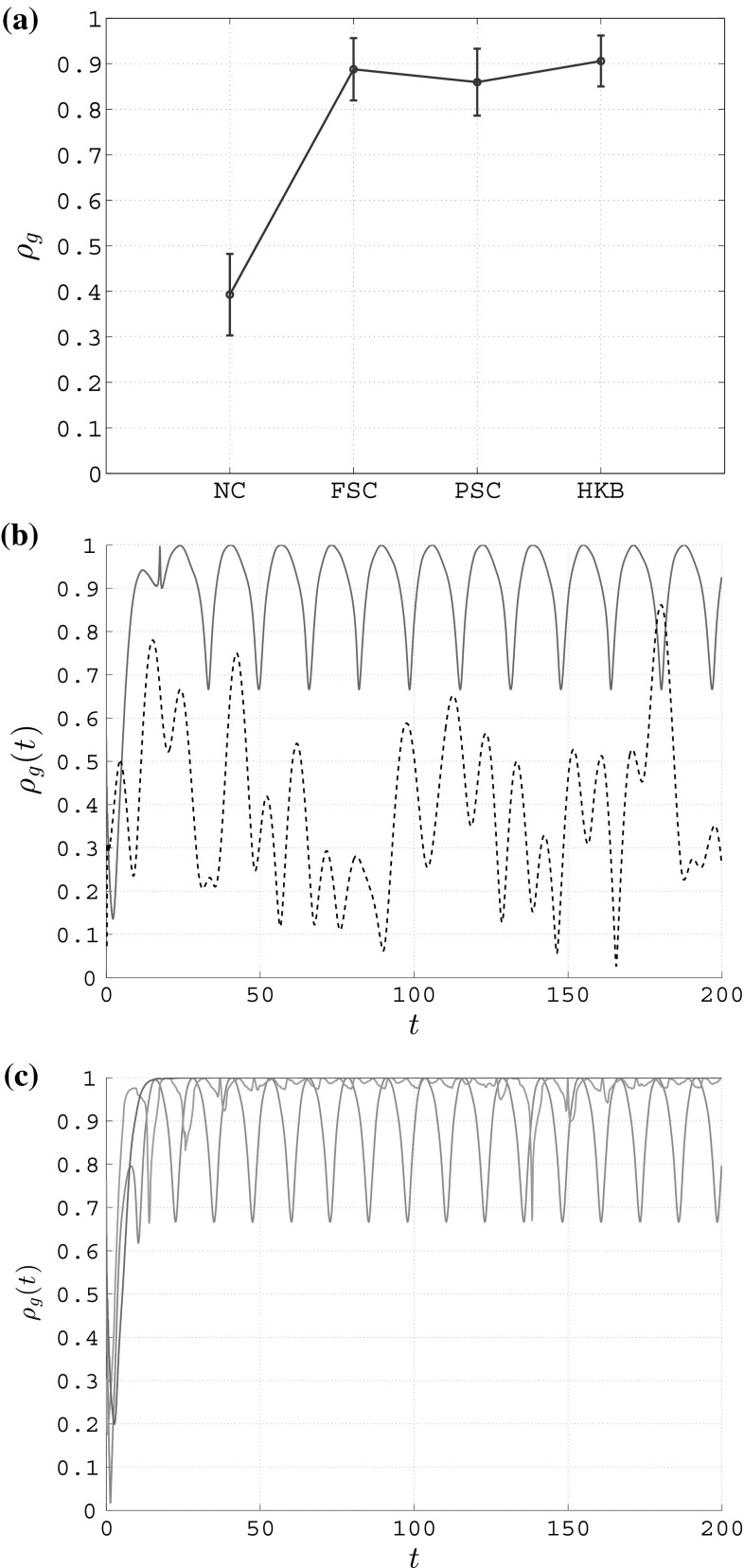
Group synchronization in an unweighted complete graph of $N=6$ heterogeneous RvdP oscillators. **a** Mean value (circle) and standard deviation (error bar); *NC* no coupling, *FSC* full state coupling ($c=0.15$), *PSC* partial state coupling ($c_1=c_2=0.15$), *HKB* HKB coupling ($a=b=-1,c=0.15$). **b** Trend over time—coupled and uncoupled scenario; *black dashed line* no coupling, *red solid line* full state coupling ($c=0.15$). **c** Trend over time—comparison among the three couplings; *red solid line* full state coupling ($c=0.17$), *gray solid line* partial state coupling ($c_1=c_2=0.17$), *light blue solid line* HKB coupling ($a=b=-1,c=0.17$)

In particular, since we are interested in replicating the key features of the rocking chairs experiments for both conditions (eyes open and eyes closed), in Fig. 2 we show the group synchronization obtained with and without coupling between the agents. In particular, in Fig. 2a we show the mean value and standard deviation of $\rho _g(t)$ for the case in which the nodes are uncoupled, and for the cases in which they are connected through each of the three coupling functions presented earlier. For each of the cases being considered, after a simulation of duration *T*, the standard deviation $\sigma _{\rho _g}$ is computed as

21 $$\begin{aligned} \sigma _{\rho _g} = \sqrt{ \frac{1}{T} \int _{0}^{T} \left( \rho _g(t)-\rho _g \right) ^2 \ {\text {d}}t }\nonumber \\ \simeq \sqrt{ \frac{1}{N_T} \sum _{i=1}^{N_T} \left( \rho _g(t_i)-\rho _g \right) ^2 } \end{aligned}$$

where $\rho _g$ is the mean value of the group synchronization index defined in Eq. 19. From Fig. 2a, it is possible to appreciate that in the absence of connections among the nodes, which corresponds to $u_i=0 \ \forall i \in [1,N]$, the group synchronization has a mean value approximately equal to 0.4, while it significantly increases (approximately 0.9) when connecting the nodes with any of the three coupling functions introduced above. These results confirm the observations previously made for a network of six human people involved in rocking chairs experiments (Fig. 1a). In particular, we heuristically chose $c=0.15$ for the full state coupling, $c_1=c_2=0.15$ for the partial state coupling, and $a=b=-1, c=0.15$ for the HKB coupling in order to match the experimental observations.

In Fig. 2b, we show the time evolution of the group synchronization index $\rho _g(t)$ when the nodes are not connected at all (black dashed line) and when they are connected through full state coupling with $c=0.15$ (red solid line): for the sake of brevity, we do not show the evolutions of $\rho _g(t)$ obtained with partial state and HKB coupling as they are similar to that obtained with full state coupling. Our model is able to reproduce another key feature observed in Richardson et al. (2012): when the nodes are uncoupled, which corresponds to the eyes closed condition, the amplitude of the oscillations of $\rho _g(t)$ is higher than that obtained when the nodes are coupled, which corresponds to the eyes open condition (Fig. 1b).

**Table 4:** Degree of synchronization of the nodes in an unweighted complete graph of $N=6$ heterogeneous RvdP oscillators—no coupling (NC), full state coupling (FSC) with $c=0.15$, partial state coupling (PSC) with $c_1=c_2=0.15$ and HKB coupling (HKB) with $a=b=-1,c=0.15$

Node	NC	FSC	PSC	HKB
1	0.42	0.95	0.93	0.97
2	0.38	0.92	0.94	0.96
3	0.45	0.98	0.95	0.97
4	0.41	0.98	0.96	0.98
5	0.33	0.33	0.33	0.49
6	0.36	0.98	0.97	0.98

However, the way a network of oscillators is coupled might in general affect the overall coordination level of the agents for certain values of the coupling strength, especially because of the different nature (linear or nonlinear) of the coupling. Indeed, when increasing the value of the coupling strength, the group synchronization $\rho _g(t)$ turns out to be qualitatively different in each of the three different cases (Fig. 2c). The oscillations observed in the rocking chairs experiments at steady state are preserved only when using partial state coupling or HKB coupling, with the latter yielding results which are more similar to those observed experimentally in Richardson et al. (2012) in terms of the oscillations amplitude.

In Table 4, we show the degree of synchronization $\rho _k$ obtained for each node of the network, both in the absence of coupling among the agents and in its presence. It is possible to appreciate how, for each node *k* in the network, $\rho _k$ has much higher values when some coupling is present, confirming what observed in Richardson et al. (2012) when contrasting synchronization levels when the participants have their eyes open or closed. Moreover, we observe that, as in the experiments, despite the group synchronization index taking higher values when the nodes are coupled, there are some agents that struggle to keep up with the general trend of the group, therefore showing lower values in terms of $\rho _k$ (node 5 in our simulations).

The fact that the degree of synchronization of node 5 is lower than that of the others can be explained by noting that its parameters are the furthest from the average values, with respect to all the other nodes in the network. In particular, if we define $\omega _i^*=\omega _i^2$ and the average values of all the parameters as follows

$$\begin{aligned} \tilde{\alpha }= & {} \frac{\sum _{i=1}^{N} \alpha _i}{N}, \tilde{\beta }=\frac{\sum _{i=1}^{N} \beta _i}{N},\\ \tilde{\gamma }= & {} \frac{\sum _{i=1}^{N} \gamma _i}{N}, \tilde{\omega }^*=\frac{\sum _{i=1}^{N} \omega _i^*}{N} \end{aligned}$$

we can evaluate their percentile error from the mean values through the following quantities:

$$\begin{aligned} \hat{\alpha }_i= & {} \frac{| \alpha _i-\tilde{\alpha } |}{\tilde{\alpha }}, \hat{\beta }_i=\frac{| \beta _i-\tilde{\beta } |}{\tilde{\beta }},\\ \hat{\gamma }_i= & {} \frac{| \gamma _i-\tilde{\gamma } |}{\tilde{\gamma }}, \hat{\omega }_i^*=\frac{| \omega _i^*-\tilde{\omega }^* |}{\tilde{\omega }^*} \end{aligned}$$

As a measure of the overall distance with respect to the average values of the parameters, we use the norm of the vector $\hat{\theta }_i = [ \hat{\alpha }_i \ \hat{\beta }_i \ \hat{\gamma }_i \ \hat{\omega }_i^* ]$. From Table 5, it is possible to appreciate that $||\hat{\theta }_5||=\max _{i \ \in [1,N]} \hat{\theta }_i$ and therefore player 5 is the furthest away from the rest of the group in parameter space.

**Table 5:** Percentile error from the average values of the parameters and overall distance for all the nodes in the network

Node	$\hat{\alpha }_i$ (%)	$\hat{\beta }_i$ (%)	$\hat{\gamma }_i$ (%)	$\hat{\omega }_i^*$ (%)	$||\hat{\theta }_i||$
1	17	16	49	68	0.87
2	6	20	61	9	0.65
3	13	72	46	54	1.02
4	57	69	63	43	1.18
5	94	24	22	144	1.75
6	36	14	51	30	0.71

**Table 6:** Dyadic synchronization in an unweighted complete graph of $N=6$ heterogeneous RvdP oscillators—full state coupling ($c=0.15$)

Nodes	2	3	4	5	6
1	0.91	0.98	0.94	0.38	0.98
2	–	0.92	0.96	0.43	0.93
3	–	–	0.97	0.39	0.99
4	–	–	–	0.40	0.98
5	–	–	–	–	0.41

**Fig. 3:**
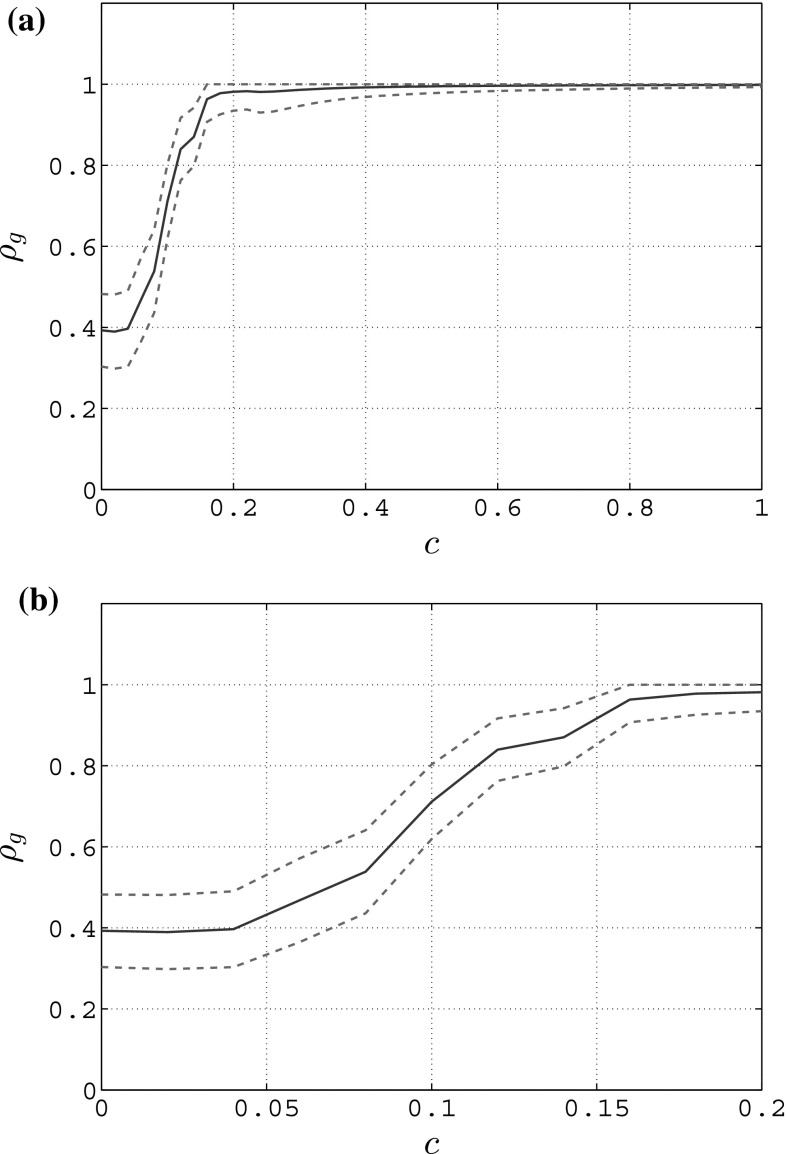
Mean value (blue solid line) and standard deviation (red dashed lines) of the group synchronization in an unweighted complete graph of $N=6$ heterogeneous RvdP oscillators for different values of the coupling strength *c*—full state coupling. **a** Group synchronization, **b** group synchronization—zoom

In Table 6, we show the dyadic synchronization $\rho _{d_{k,h}}$ for all the possible couples of nodes in the network: again, our simulation results confirm what observed for the rocking chairs experiments. Indeed, the pairs of nodes with lower dyadic synchronization correspond to pairs in which at least one of the two nodes had trouble synchronizing with the general trend of the group (node 5 in our simulations). For the sake of clarity, we show $\rho _{d_{k,h}}$ only when connecting the nodes through full state coupling. Analogous results can be obtained also for the other two strategies introduced earlier in this paper.

It is easy to foresee that, regardless of the coupling function the nodes are connected through, the value of the coupling strength has a direct impact on the group synchronization in terms of its mean value and its standard deviation. We now show how $\rho _g$ varies quantitatively as the coupling strength varies for all the three coupling functions introduced earlier in this paper, when considering an unweighted complete graph of $N=6$ heterogeneous RvdP oscillators whose parameters and initial values are those given in Table 3. Moreover, we once again set $T=200$ s.

**Fig. 4:**
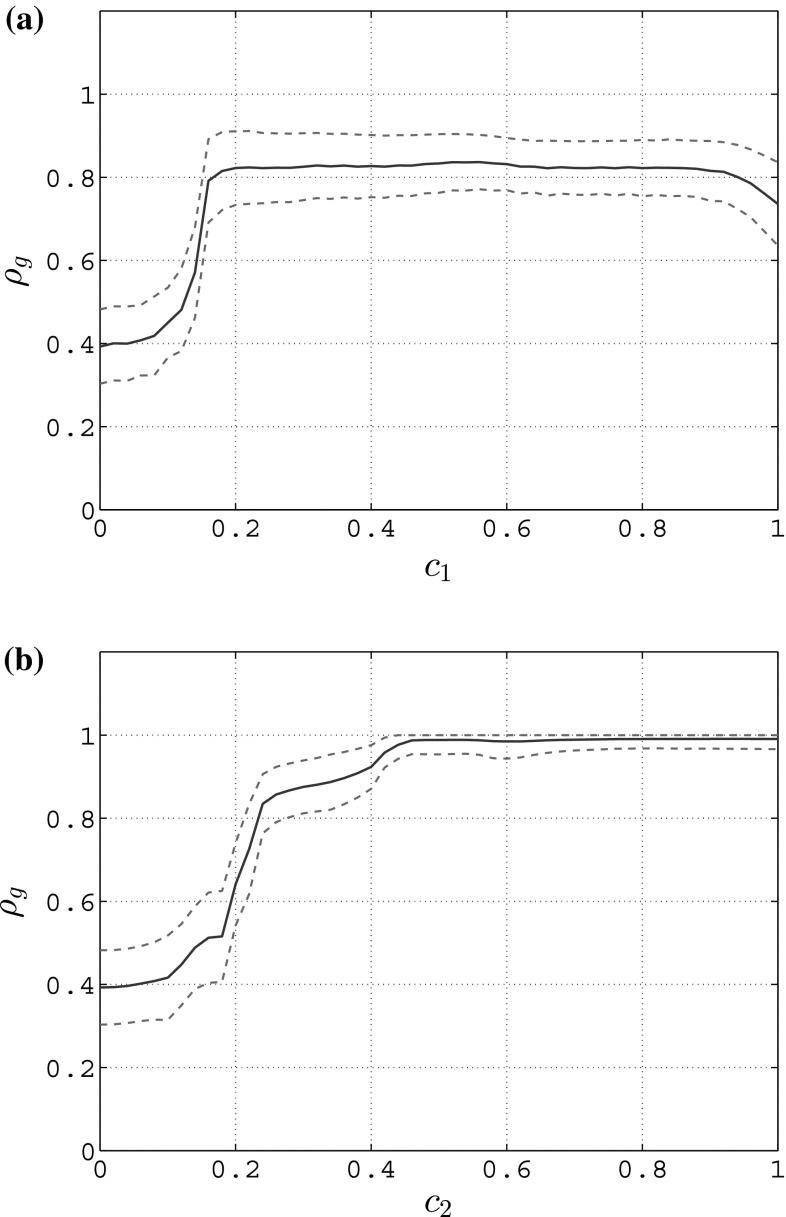
Mean value (blue solid line) and standard deviation (red dashed line) of the group synchronization in an unweighted complete graph of $N=6$ heterogeneous RvdP oscillators for different values of the coupling strengths $c_1$ and $c_2$—partial state coupling. **a** $c_2=0, c_1$ variable, **b** $c_1=0, c_2$ variable

**Fig. 5:**
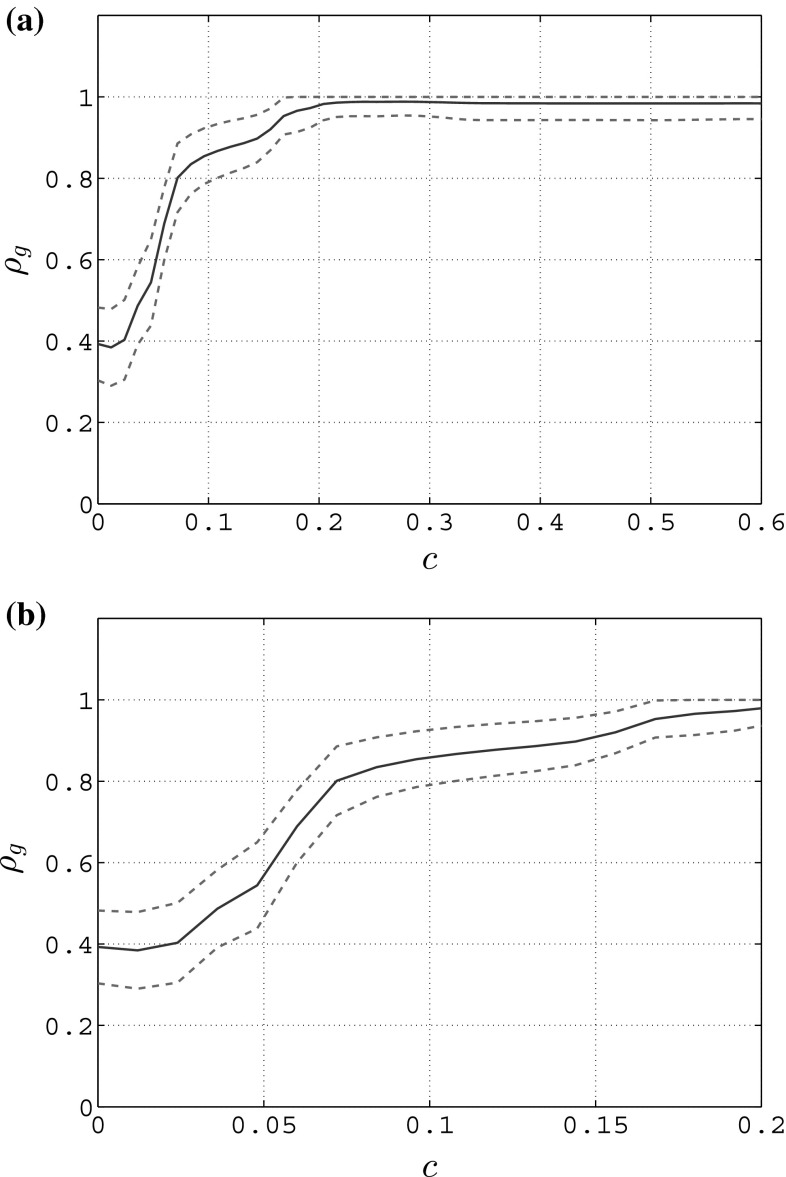
Mean value (blue solid line) and standard deviation (red dashed line) of the group synchronization in an unweighted complete graph of $N=6$ heterogeneous RvdP oscillators for different values of the coupling strength *c* while keeping $a=b=-1$ constant—HKB coupling. **a** Group synchronization, **b** group synchronization—zoom

In Figs. 3, 4 and 5, we show the mean value and standard deviation of the group synchronization index $\rho _g(t)$ obtained for different values of the coupling strength when considering full state coupling, partial state coupling and HKB coupling.

From Fig. 3a, it is clear that, when considering full state coupling, the group synchronization index increases as the coupling strength *c* increases: in particular, a relatively small value of the coupling strength is sufficient for the network to synchronize within an acceptable bound ($c \simeq 0.15$ leads to $\rho _g \simeq 0.9$, see Fig. 3b). This means that the stronger the influence of each player on the others is, the better the overall synchronization of the human participants.

**Fig. 6:**
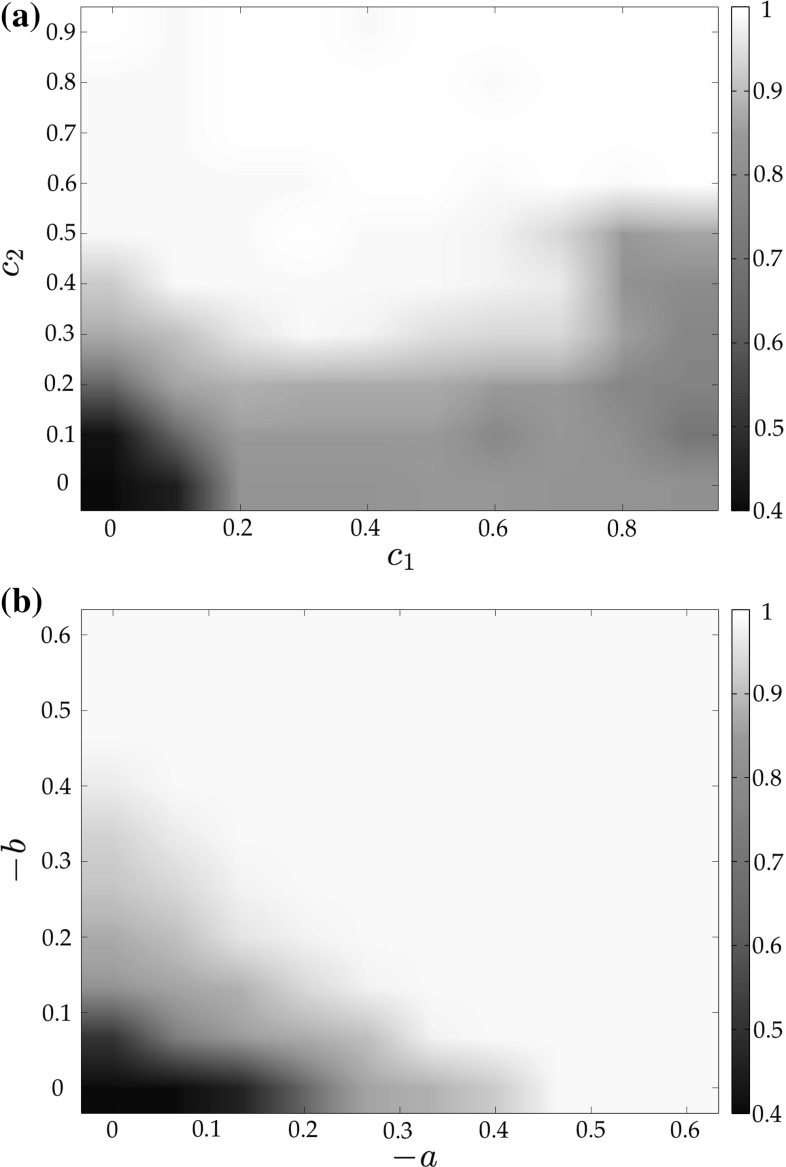
Mean value of the group synchronization index $\rho _g(t)$ in an unweighted complete graph of $N=6$ heterogeneous RvdP oscillators for different values of the coupling strengths. Darker colors refer to lower values of the average group synchronization, while lighter ones correspond to higher values. **a** Partial state coupling—$c_1,c_2$ variable, **b** HKB coupling—*a*, *b* variable while keeping $c=1$ constant

In Fig. 4a, we show how, when considering a partial state coupling, the group synchronization index varies for increasing values of $c_1$ while keeping $c_2$ constant and equal to 0, and vice versa in Fig. 4b. It is possible to appreciate how the influence that $c_2$ has on the group synchronization is stronger than that provided by $c_1$. A possible interpretation of this effect is that human players react better to changes in the velocity of their neighbors rather than to changes in their position. This result is also confirmed in terms of the mean value of the group synchronization index as $c_1$ and $c_2$ are simultaneously varied (Fig. 6a).

Finally from Fig. 5a it is clear that, when considering HKB coupling while keeping *a* and *b* constant and equal to $-1$, the group synchronization increases as the coupling strength *c* increases. In particular, like in the case of full state coupling, in order for the network to well synchronize it is sufficient to choose a relatively small value for the coupling strength ($c \simeq 0.15$ leads to $\rho _g \simeq 0.9$, see Fig. 5b). A possible interpretation of this effect is that the stronger the influence that each player has on the others, the better the overall synchronization of the human participants. This result is confirmed also in Fig. 6b in which we show how the mean value of the group synchronization changes as *a* and *b* are simultaneously varied while keeping *c* constant and equal to 1. It is possible to appreciate how, as the values of |*a*| and |*b*| increase, then so does the average of the group synchronization index.

## Entrainment of the network

In this section, we analyze the effects on the group synchronization of an entrainment signal affecting all nodes, modeled by adding an external sinusoidal signal to their dynamics. Our main objective is understanding whether, and possibly under what conditions, such entrainment signal leads to better coordination level in a network of heterogeneous RvdP oscillators when compared to the case in which the signal is absent. This will help us uncover whether the presence of an external auditory or visual stimulus can improve synchronization in a group of individuals performing some joint coordination task.

Following the approach of Russo et al. (2010), we model such a scenario in the following way:

22 $$\begin{aligned} \dot{x}_i= \begin{bmatrix} x_{i_2} \\ - (\alpha _i x_{i_1}^2+\beta _i x_{i_2}^2-\gamma _i)x_{i_2} - \omega _i^2 x_{i_1} + \zeta \end{bmatrix}+u_i \end{aligned}$$

where $\zeta (t)=A_\zeta \sin \left( \omega _\zeta t \right)$ represents the entrainment signal and $u_i(t)$ one of the coupling functions introduced earlier in this paper.

We introduce the *entrainment index*$\rho _E \in [0,1]$ in order to quantify the overall coordination level between the network and the external signal $\zeta (t)$:

23 $$\begin{aligned} \rho _{E} := \frac{1}{N} \sum _{k=1}^{N} \rho _{E_k} \end{aligned}$$

where $\rho _{E_k} := \left| \frac{1}{T} \int _{0}^{T} e^{ j [ \theta _k(t)- \theta _\zeta (t) ] } \ {\text {d}}t \right| ,\theta _k(t)$ is the phase of the *k*th node, $\theta _\zeta (t)$ is the phase of $\zeta (t)$, *T* is the duration of the experiment, and *N* is the number of nodes in the network. The closer $\rho _E$ is to 1, the stronger the entertainment of the group with the external signal.

The simulation scenario is the same as that described in Sect. 5.1, with the addition of the entrainment signal.

**Fig. 7:**
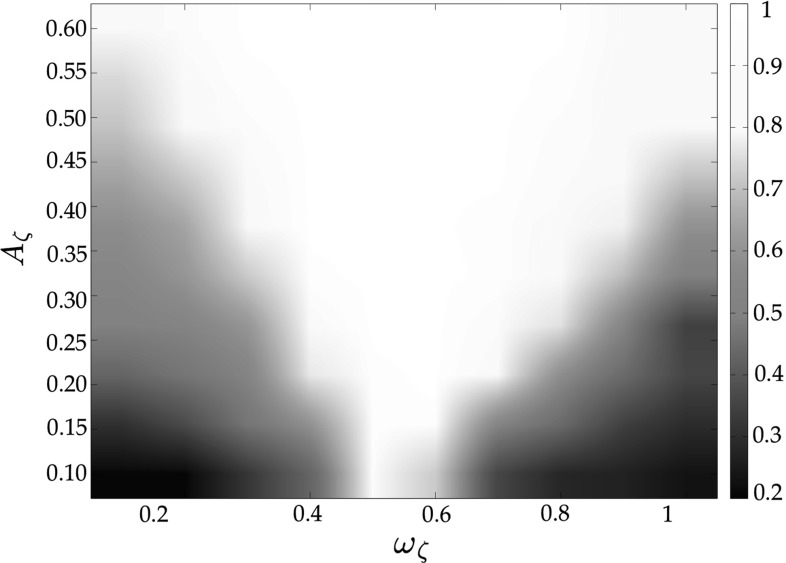
Entrainment index in an unweighted complete graph of $N=6$ heterogeneous RvdP oscillators—full state coupling ($c=0.15$). Darker colors refer to lower values of the index, while lighter ones denote higher values

In Fig. 7, we show the values of the entrainment index for different values of the frequency $\omega _\zeta$ and the amplitude $A_\zeta$ of the entrainment signal $\zeta (t)$ when considering full state coupling with $c=0.15$. It is possible to appreciate how, for each value of $\omega _\zeta$, the entrainment index increases as $A_\zeta$ increases, meaning that the network better synchronizes with $\zeta (t)$ for increasing values of its amplitude. Moreover, for a given value of $A_\zeta$, the highest values of $\rho _E$ are achieved when the frequency of the entrainment signal is close to the average value ${\varOmega }$ of the natural frequencies $\omega _i$ of the nodes (in this case ${\varOmega } \simeq 0.5$ ). These results confirm the findings of Schmidt et al. (2007); Varlet et al. 2015), in which it is shown that spontaneous unintentional synchronization between the oscillation of a handheld pendulum swung by an individual and an external sinusoidal stimulus (which corresponds to our external entrainment signal) emerges only when the frequency of the signal itself is similar to the preferred frequency of the player. For the sake of brevity, we do not show how $\rho _E$ varies as $\omega _\zeta$ and $A_\zeta$ vary when considering partial state coupling and HKB coupling, since we obtain results which are similar to those shown in Fig. 7 for full state coupling.

**Fig. 8:**
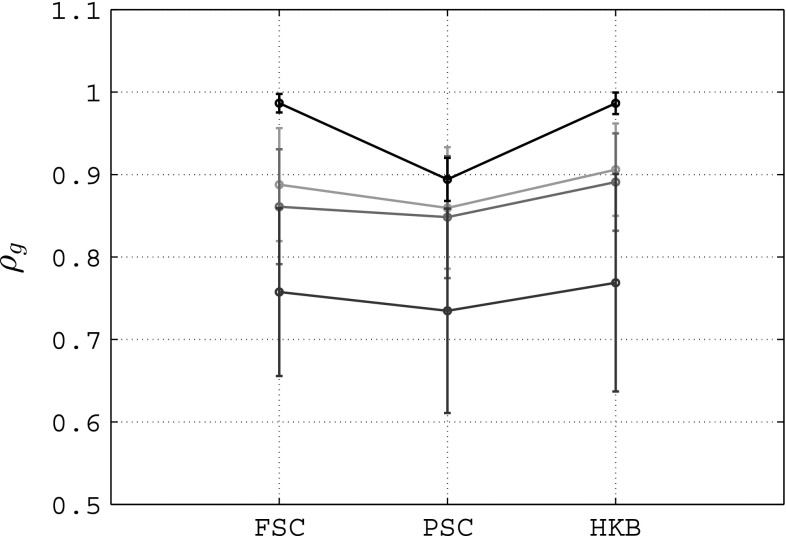
Mean value (circle) and standard deviation (error bar) of the group synchronization in an unweighted complete graph of $N=6$ heterogeneous RvdP oscillators. *FSC* full state coupling ($c=0.15$), *PSC* partial state coupling ($c_1=c_2=0.15$), *HKB* HKB coupling ($a=b=-1, c=0.15$); *green line* no entrainment signal, *red line* $\omega _\zeta =0.1, A_\zeta =0.1$, *blue line* $\omega _\zeta =0.3, A_\zeta =0.2$, *black line* $\omega _\zeta =0.5, A_\zeta =0.3$

In Fig. 8, we show the mean value and standard deviation of the group synchronization index $\rho _g(t)$ when considering different parameters of the entrainment signal for all the three coupling functions we have presented. As we are interested in understanding whether an additive external sinusoidal signal can improve the coordination level of the network, the values of the coupling strengths chosen in these simulations for all the three coupling functions are the same as those previously used in the absence of entrainment signal (Fig. 2a). From Fig. 8, it is easy to observe that, for all coupling functions, the group synchronization index of the network improves only when the entrainment index $\rho _E$ has high values (see black line compared to the green one). In the other two cases (blue line and red line), the entrainment signal acts as a disturbance for the dynamics of the nodes and the group synchronization index decreases. This confirms that it is possible to further enhance the coordination level of participants only when the entrainment signal has an oscillation frequency which is close to the average of the natural oscillation frequencies of the individuals involved and its amplitude is sufficiently high.

## Convergence analysis

As anticipated earlier, we are also interested in understanding under what conditions synchronization is theoretically observed to emerge. In particular, in this section we are going to show that bounded synchronization can be analytically guaranteed for a network of *N* diffusively coupled non-identical RvdP oscillators by making use of two different approaches, namely *contraction theory* and *Lyapunov theory*.

### Contraction theory

Let $|\cdot |$ be a norm defined on a vector $w \in {\mathbb {R}}^n$ with induced matrix norm $||\cdot ||$. As stated in Russo et al. (2013), given a matrix $P \in {\mathbb {R}}^{n \times n}$, the *induced matrix measure* is defined as $\mu (P) := \lim _{h \rightarrow 0^+} \frac{\left( ||I+hP|| -1 \right) }{h}$.

#### **Definition 3**

Let us consider the system $\dot{w} = F(t,w)$ defined $\forall t \ge 0, \ \forall w \in C \subset {\mathbb {R}}^n$. We say that such system is contracting with respect to a norm $|\cdot |$ with associated matrix measure $\mu (\cdot )$ iff

24 $$\begin{aligned} \exists \ k>0: \mu \left( J(w,t) \right) \le -k, \quad \forall w \in C, \forall t \ge 0 \end{aligned}$$

where *J* is the Jacobian of the system.

The key stage in the application of contraction theory to synchronization of networks of oscillators is the construction of the so-called *virtual system* (Jouffroy and Slotine 2004).

#### **Definition 4**

Let us consider a network of identical systems. The virtual system is defined as a dynamical system expressed in some auxiliary variable set whose particular solutions are the trajectories of each of the nodes in the network.

Formally, the virtual system depends on the state variables of the agents in the network and on some virtual state variables. Substitution of the state variables of a certain node *i* with the virtual ones returns the dynamics of the *i*th node of the network itself. Then, if the virtual system is contracting, complete synchronization is achieved (Russo and di Bernardo 2009; Wang and Slotine 2005 ; di Bernardo et al. 2016).

As noted in Russo and di Bernardo (2009), virtual systems can also be used to study networks of oscillators with parameter mismatches. Indeed, it is shown that if a network of identical systems achieves complete synchronization, then it achieves bounded synchronization when parameter mismatches among the nodes are present (see Russo and di Bernardo 2009 for further details and example of application to networks of heterogeneous biological oscillators).

In Russo and di Bernardo (2009), a simple algorithm is provided that allows to check whether the virtual system of a certain network of *N* non-identical agents is contracting, which leads to bounded synchronization of the network itself. In particular, rather than verifying Eq. 24 in order to check whether the virtual system is contracting, the algorithm consists in checking the truth of some statements regarding the single elements of the Jacobian of the virtual system and imposing some conditions:

1. build the Jacobian *J* of the virtual system;
2. check whether the following statements are true or false
  S1: $$J_{ii}<0$$;
  S2: $$J_{ii}=-\rho _i, \ 0<\rho _i<\infty $$;
  S3: $$J_{ij}\ne 0 \Rightarrow J_{ji}=0$$;
3. generate a set of conditions for synchronization (CFS) according to the truth or the falsity of the previous statements.

In particular, denoting with $n_{0_i}$ the number of 0 elements in the *i*th row of the Jacobian of the virtual system, the CFS are generated in the following way:

- $$S1, S2, S3 \Rightarrow |J_{ij}|<\frac{\rho _i}{n-n_{0_i}-1}$$ ;
- $S1, S2, \bar{S3} \Rightarrow |J_{ij}|>\frac{\rho _i}{n-n_{0_i}-1}, \ |J_{ji}|<\frac{\rho _j}{n-n_{0_j}-1}$ or vice versa;
- $$S1, \bar{S2}, S3 \Rightarrow |J_{ij}|<\frac{|J_{ii}|}{n-n_{0_i}-1}$$;
- $S1, \bar{S2}, \bar{S3} \Rightarrow |J_{ij}|>\frac{|J_{ii}|}{n-n_{0_i}-1}, \ |J_{ji}|<\frac{|J_{jj}|}{n-n_{0_j}-1}$ or vice versa.

Note that if statement *S1* is not true, it is not possible for the virtual system to be contracting.

#### **Theorem 1**

Suppose to have a network of *N* heterogeneous RvdP oscillators interconnected via full state coupling as described in Eq. 5. Let us also assume that the network topology is a connected, simple, undirected, and unweighted complete graph. If the following hypothesis of inequalities is satisfied

25 $$\begin{aligned} \frac{N-1}{N} \left( 2\tilde{\alpha }z_{1_{\max }}z_{2_{\max }}+\tilde{\omega }^2+\tilde{\gamma } \right) < c < \frac{N-1}{N} \end{aligned}$$

where $\tilde{\alpha }, \tilde{\omega }, \tilde{\gamma }$ are the average values of parameters $\alpha _i$, $\omega _i$, $\gamma _i$, respectively, and $z_{1_{\max }}, z_{2_{\max }}$ are the bounds of the two virtual state variables introduced below in Eq. 27, then bounded synchronization is achieved by the network.

#### *Proof*

Let us consider an unweighted complete graph of *N* heterogeneous RvdP oscillators interconnected via full state coupling, that is

26 $$\begin{aligned} \dot{x}_i= & {} \begin{bmatrix} x_{i_2} \\ - (\alpha _i x_{i_1}^2+\beta _i x_{i_2}^2-\gamma _i)x_{i_2} - \omega _i^2 x_{i_1} \end{bmatrix}\nonumber \\&-\,\hat{c} \sum _{j=1}^N a_{ij} \left( x_i-x_j \right) , \quad \forall i \in [1,N] \end{aligned}$$

where $x_i \in {\mathbb {R}}^2$ is the state variable of node *i* and $\hat{c} := \frac{c}{N-1}$ since each node in a connected complete graph has $N-1$ neighbors. The virtual system corresponding to this network can be written as

27 $$\begin{aligned} \dot{z}= & {} \begin{bmatrix} z_2\\ -\left( \tilde{\alpha }z_1^2+\tilde{\beta }z_2^2-\tilde{\gamma } \right) z_2 - \tilde{\omega }^2 z_1 \end{bmatrix}\nonumber \\&- \begin{bmatrix} \hat{c}Nz_1 - \hat{c}\sum _{j=1}^{N} x_{j_1} \\ \hat{c}Nz_2 - \hat{c} \sum _{j=1}^{N} x_{j_2} \end{bmatrix} \end{aligned}$$

where $z \in {\mathbb {R}}^2$ is the vector of auxiliary variables of the virtual system and $\tilde{\alpha }:=\frac{1}{N}\sum _{i=1}^{N} \alpha _i$, $\tilde{\beta }:=\frac{1}{N}\sum _{i=1}^{N} \beta _i$, $\tilde{\gamma }:=\frac{1}{N}\sum _{i=1}^{N} \gamma _i$, $\tilde{\omega }:=\frac{1}{N}\sum _{i=1}^{N} \omega _i$ indicate the average values of all the different parameters of the oscillators in the heterogeneous network. The Jacobian of the virtual system is:

28 $$\begin{aligned} J(t,z) = \begin{bmatrix} -\hat{c}N&1 \\ -(2\tilde{\alpha }z_2z_1+\tilde{\omega }^2)&\quad -\tilde{\alpha }z_1^2-3\tilde{\beta }z_2^2-\hat{c}N+\tilde{\gamma } \end{bmatrix}\nonumber \\ \end{aligned}$$

In order to prove bounded synchronization of the network, we need the virtual system to be contracting. In order to do so, we apply the algorithm presented in Russo and di Bernardo (2009) to Eq. 28. When $i=1,j=2$, it is immediate to see that statement *S1* is true, while *S2* and *S3* are false (*c* might be in general time varying), leading to $|J_{12}|>|J_{11}|$ and $|J_{21}|<|J_{22}|$. When $i=2,j=1$ instead, inequalities to satisfy and the associated CFS depend on the sign of $\tilde{\alpha }$ and $\tilde{\beta }$. Supposing without loss of generality that $\tilde{\alpha },\tilde{\beta }>0$ as usually done in the literature (Fuchs and Jirsa 2008; Kelso et al. 2009), it is immediate to see that an inequality corresponding to the fulfillment of *S1* needs to be added to the list of CFS generated by the algorithm (a worst-case scenario is $-\hat{c}N+\tilde{\gamma }<0$) and that both *S2* and *S3* are again false, leading to the two same conditions. This means that the network achieves bounded synchronization when the following system of inequalities is satisfied:

29 $$\begin{aligned}&{\left\{ \begin{array}{ll} \hat{c}>\frac{\tilde{\gamma }}{N}\\ 1>\hat{c}N\\ | 2\tilde{\alpha }z_1z_2+\tilde{\omega }^2 | < | \tilde{\alpha }z_1^2+3\tilde{\beta }z_2^2-\tilde{\gamma }+\hat{c}N | \end{array}\right. }\nonumber \\&\Leftrightarrow {\left\{ \begin{array}{ll} \frac{\tilde{\gamma }}{N}<\hat{c}<\frac{1}{N}\\ | 2\tilde{\alpha }z_1z_2+\tilde{\omega }^2 | < | \tilde{\alpha }z_1^2+3\tilde{\beta }z_2^2-\tilde{\gamma }+\hat{c}N | \end{array}\right. } \end{aligned}$$

Supposing that the dynamics of the virtual system is bounded, meaning that $$|z_1(t)| \le z_{1_{\max }}, |z_2(t)| \le z_{2_{\max }}$$$$\forall t \ge 0$$, we can consider the following worst-case scenario

30 $$\begin{aligned} {\left\{ \begin{array}{ll} \frac{\tilde{\gamma }}{N}<\hat{c}<\frac{1}{N}\\ 2\tilde{\alpha }z_{1_{\max }}z_{2_{\max }}+\tilde{\omega }^2 < \hat{c}N - \tilde{\gamma } \end{array}\right. } \end{aligned}$$

which, since $\hat{c}=\frac{c}{N-1}$, yields Eq. 25.

So we can conclude that if the coupling strength *c* fulfills Eq. 25, the network of heterogeneous RvdP oscillators overlying a complete graph achieves bounded synchronization. $\square$

#### *Remark 2*

Note that when the number of nodes in the network *N* tends to infinity, then $\frac{N-1}{N} \rightarrow 1$. This means that bounded synchronization is achieved when:

31 $$\begin{aligned} 2\tilde{\alpha }z_{1_{\max }}z_{2_{\max }}+\tilde{\omega }^2+\tilde{\gamma } < c < 1. \end{aligned}$$

### Lyapunov theory

Let ${\mathcal {D}}$ be the set of diagonal matrices, ${\mathcal {D}}^+$ the set of positive definite diagonal matrices, and ${\mathcal {D}}^-$ the set of negative definite diagonal matrices. Let us now define *QUAD* and *QUAD Affine* vector fields (DeLellis et al. 2015).

#### **Definition 5**

Given $n \times n$ matrices $P \in {\mathcal {D}}^+, W_i \in {\mathcal {D}}$, the vector field $f_i$ is said to be *QUAD*($P,W_i$) iff

32 $$\begin{aligned} (z-w)^T P [f_i(t,z)-f_i(t,w)] \le (z-w)^T W_i (z-w)\nonumber \\ \end{aligned}$$

for any $z,w \in {\mathbb {R}}^n$ and for any $t \ge 0$.

#### **Definition 6**

Given $n \times n$ matrices $P \in {\mathcal {D}}^+, W_i \in {\mathcal {D}}$, the vector field $f_i$ is said to be *QUAD*($P,W_i$) *Affine* iff $f_i(t,x_i)=h_i(t,x_i)+g_i(t,x_i)$ and

- $$h_i$$ is QUAD($$P,W_i$$);
- $$\exists \ M<\infty : \text {such that} ||g_i(t,z)||_2<M, \ \forall z {\in } {\mathbb {R}}^n, \forall t {\ge } 0$$

Let us consider a network of *N* non-identical agents interconnected via linear coupling:

33 $$\begin{aligned} \dot{x}_i(t) = f_i(t,x_i)-\frac{c}{{\mathcal {N}}_i}\sum _{j=1}^{N}a_{ij}{\varGamma }(x_i-x_j), \quad c>0 \end{aligned}$$

where ${\varGamma } \in {\mathbb {R}}^{n \times n}$. Note that this is a generalization of the full state coupling previously introduced, which can be obtained by setting ${\varGamma }=I_n$. As reported in DeLellis et al. (2015) in details, in order to prove bounded synchronization for a network of *N* non-identical QUAD Affine systems interconnected via a linear coupling function, we need $h_i(t,x_i)$ to be QUAD($P,W_i$) with $W_i \in {\mathcal {D}}^-$ for all the nodes in the network at any time instant. However, in a network of *N* heterogeneous RvdP oscillators with vector fields described by Eq. 4, regardless of the way we define $h_i$ and $g_i$ it is never possible to satisfy the following condition

34 $$\begin{aligned} (z-w)^T P [h_i(t,z)-h_i(t,w)] \le (z-w)^T W_i (z-w)\nonumber \\ \end{aligned}$$

with definite negative matrices $W_i$. Indeed, the right-hand side is always negative, while the left-hand side can be positive for any value of $P>0$. On the other hand, in order to avoid conditions on the sign of the matrices $W_i$, it is necessary to write the dynamics of the nodes in the following way

35 $$\begin{aligned} f_i(t,x_i)=h_i(t,x_i)+g_i(t,x_i) \quad \forall i=1,2,\ldots ,N \end{aligned}$$

with $h_i(t,z)=h_j(t,z)=h(t,z) \ \forall i,j \in [1,N], \ \forall z \in {\mathbb {R}}^n$, and with all the terms $g_i$ being bounded, at any time instant. In particular, in DeLellis et al. (2015) the authors prove the following theorem.

#### **Theorem 2**

Let us consider a network of *N* non-identical agents interconnected via linear coupling as described in Eq. 33. Let us suppose that $f_i(t,x_i)=h(t,x_i)+g_i(t,x_i)$ and that:

1. the network is made up of *N* QUAD(*P*, *W*) Affine systems, with $P \in {\mathcal {D}}^+$ and $W \in {\mathcal {D}}$;
2. ${\varGamma }$ is a positive semidefinite diagonal matrix;
3. if *W* is made up of  $l \in [0,n]$ nonnegative elements, which without loss of generality can be collected in its $l \times l$ upper-left block, then ${\varGamma }$ is made up of  $\bar{l}$ positive elements, where $l \le \bar{l} \le n$, which again without loss of generality can be collected in its $\bar{l} \times \bar{l}$ upper-left block;
4. $$\exists \ 0<\bar{M}<\infty $$ such that $$||g_i(t,x_i)||_2<\bar{M} \ \forall i=1,2,\ldots ,N, \forall t \ge 0$$.

Then, bounded synchronization is achieved by the network. In particular, if we define the matrix $L_{{\mathcal {N}}}=\{ l_{{\mathcal {N}}_{ij}} \}$ as

36 $$\begin{aligned} l_{{\mathcal {N}}_{ij}} := {\left\{ \begin{array}{ll} \frac{1}{{\mathcal {N}}_i} \sum _{k=1}^N a_{ik}, &{} \text{ if } i=j \\ -\frac{a_{ij}}{{\mathcal {N}}_i}, &{} \text{ if } i \ne j \text{ and } (i,j) \text{ are } \text{ neighbors }\\ 0, &{} \text{ otherwise } \end{array}\right. }\nonumber \\ \end{aligned}$$

we can state that $\exists \ 0<\bar{c}<\infty , {\epsilon }>0$ such that $\lim _{t \rightarrow \infty } \eta (t) \le {\epsilon } \ \forall c > \bar{c}$, where

37 $$\begin{aligned} \bar{c} = \min _{P,W} \ \max \left( \frac{\lambda _M\left( W_l \right) }{\lambda _2 \left( L_{{\mathcal {N}}} \otimes P_l {\varGamma }_l \right) },0 \right) \end{aligned}$$

with $W_l,P_l,{\varGamma }_l$ representing the $l \times l$ upper-left block of matrices $W,P,{\varGamma }$, respectively, and where for a given value of $c>\bar{c}$

38 $$\begin{aligned} {\epsilon } = \min _{P,W} \ \frac{-\sqrt{N} \bar{M} ||P||_2}{\max \left( \lambda _M\left( W_l \right) -c \lambda _2 \left( L_{{\mathcal {N}}} \otimes P_l {\varGamma }_l \right) , \lambda _M \left( W_{n-l} \right) \right) }\nonumber \\ \end{aligned}$$

with $W_{n-l}$ representing the $(n-l) \times (n-l)$ lower-right block of matrix *W* and with the assumption that $c \lambda _2 \left( L_{{\mathcal {N}}} \otimes P_l {\varGamma }_l \right) > \lambda _M\left( W_l \right)$.

#### *Proof*

See DeLellis et al. (2015). $\square$

We can thus derive the following corollary.

#### **Corollary 1**

Let us consider a network of *N* heterogeneous RvdP oscillators interconnected via full state coupling. Supposing that the topology of the network is simple and undirected, and assuming that $\gamma _i=\tilde{\gamma } \ \forall i \in [1,N]$, if the coupling strength satisfies the inequality

39 $$\begin{aligned} c \ge \bar{c} = \min _{W_{11},P_{11},P_{22}>0} \ \frac{\max \left( W_{11},\tilde{\gamma } P_{22} \right) }{\lambda _2\left( L_{{\mathcal {N}}} \right) \min _{j=1,2}\left( P_{jj}{\varGamma }_{jj} \right) } \end{aligned}$$

then bounded synchronization is achieved by the network. In particular, we can claim that

40 $$\begin{aligned} \lim _{t \rightarrow \infty } \eta (t) \le {\epsilon } = \min _{W_{11},P_{11},P_{22},d_{\epsilon }>0} \ \frac{\sqrt{N}\bar{M} \max \left( P_{11},P_{22} \right) }{d_{\epsilon }}\nonumber \\ \end{aligned}$$

where

41 $$\begin{aligned} d_{\epsilon }:=c\lambda _2 \left( L_{{\mathcal {N}}} \right) \min _{j=1,2 } \left( P_{jj} {\varGamma }_{jj} \right) -\max \left( W_{11},\tilde{\gamma } P_{22} \right) \end{aligned}$$

#### *Proof*

First of all we need to write the dynamics of each node in the network as $f_i(t,x_i)=h(t,x_i)+g_i(t,x_i)$. This is possible if we suppose that $\gamma _i=\tilde{\gamma } \ \forall i \in [1,N]$ and define:

$$\begin{aligned} h(t,x_i)= & {} \begin{bmatrix} 0\\ \tilde{\gamma } x_{i_2} \end{bmatrix}\\ g_i(t,x_i)= & {} \begin{bmatrix} x_{i_2}\\ -(\alpha _i x_{i_1}^2 + \beta _i x_{i_2}^2)x_{i_2}-\omega _i^2x_{i_1} \end{bmatrix} \end{aligned}$$

Then we need to verify whether the nodes in the network are QUAD(*P*, *W*) Affine systems. In particular, this means that we need *h* to be QUAD(*P*, *W*), with $P \in {\mathcal {D}}^+$ and $W \in {\mathcal {D}}$. Therefore, if we define $P= \text {diag} \{P_{11},P_{22} \}$ with $P_{11},P_{22}>0$, $W=\text {diag} \{W_{11},W_{22} \}$ and $h(t,z)=[0 \ \ \tilde{\gamma } z_2]^T \ \forall z \in {\mathbb {R}}^2$, we have to satisfy:

42 $$\begin{aligned} P_{22} \tilde{\gamma } (z_2-w_2)^2 \le W_{11}(z_1-w_1)^2+W_{22}(z_2-w_2)^2 \end{aligned}$$

Choosing $W_{22}=\tilde{\gamma } P_{22}$, it possible to reduce Eq. 42 to

43 $$\begin{aligned} W_{11}(z_1-w_1)^2 \ge 0 \end{aligned}$$

which is true for any $W_{11}>0$. This means that the first hypothesis of Theorem 2 simply reduces to choosing any $P \in {\mathcal {D}}^+$ and $W=\text {diag} \{ W_{11},\tilde{\gamma } P_{22} \}$ for any $W_{11}>0$.

Since $W \in {\mathbb {R}}^{2 \times 2}$ is made up of 2 nonnegative elements, we have that $l=\bar{l}=2$. Therefore, in order to satisfy the second and the third hypotheses of Theorem 2, we need ${\varGamma }$ to be a diagonal positive definite matrix, that is ${\varGamma } \in {\mathcal {D}}^+$ (note that this is true since the nodes are connected through full state coupling, and hence ${\varGamma }=I_2$).

Finally, the last hypothesis left to satisfy is related to the boundedness of the terms $g_i$ at any time instant. As already shown before, we have chosen:

44 $$\begin{aligned} g_i(t,x_i) = \begin{bmatrix} x_{i_2}\\ -(\alpha _i x_{i_1}^2 + \beta _i x_{i_2}^2)x_{i_2}-\omega _i^2x_{i_1} \end{bmatrix} \end{aligned}$$

Since the dynamics of each RvdP oscillator is bounded (Zhai et al. 2015c), we can define

$$\begin{aligned} p_{i_{\max }} := \sup _{t \ge 0} \left( |x_{i_1}(t)| \right) , \ v_{i_{\max }} := \sup _{t \ge 0} \left( |x_{i_2}(t)| \right) \end{aligned}$$

and $p_M := \max _{i} \left( p_{i_{\max }} \right)$, $v_M := \max _{i} \left( v_{i_{\max }} \right)$, $\alpha _M := \max _{i} \left( |\alpha _i| \right)$, $\beta _M := \max _{i} \left( |\beta _i| \right)$, $\omega _M := \max _{i} \left( |\omega _i| \right)$. Therefore, from Eq. 44 we get:

45 $$\begin{aligned}&||g_i||_2 \le |x_{i_2}| + |(\alpha _i x_{i_1}^2 + \beta _i x_{i_2}^2)x_{i_2}+\omega _i^2x_{i_1}|\nonumber \\&\quad \le |x_{i_2}| + |\alpha _i x_{i_1}^2 + \beta _i x_{i_2}^2| |x_{i_2}| + \omega _i^2 |x_{i_1}|\nonumber \\&\quad \le (1+|\alpha _i| p_{i_{\max }}^2 + |\beta _i| v_{i_{\max }}^2) v_{i_{\max }} + \omega _i^2 p_{i_{\max }} := M_i\nonumber \\ \end{aligned}$$

Besides, we have that

46 $$\begin{aligned} M_i \le (1+\alpha _M p_M^2 + \beta _M v_M^2) v_M + \omega _M^2 p_M := \bar{M} \end{aligned}$$

This means that the fourth hypothesis of Theorem 2 is always satisfied in the case of RvdP oscillators, and the bound $\bar{M}$ is defined in Eq. 46.

In order to find an easier expression of the minimum value required for the coupling strength and of the upper bound for the error norm, we can take advantage of the particular form of matrices *P* and *W*:

$$\begin{aligned} P= & {} P_2 = \begin{bmatrix} P_{11}&0 \\ 0&P_{22} \end{bmatrix}, \qquad P_{11},P_{22}>0\\ W= & {} W_2 = \begin{bmatrix} W_{11}&0\\ 0&\tilde{\gamma }P_{22} \end{bmatrix}, \qquad W_{11}>0 \end{aligned}$$

Therefore, from Eq. 37 we have that the minimum value $\bar{c}$ for the coupling strength that guarantees bounded synchronization of the network is given by Eq. 39, while from Eq. 38 we have that the upper bound of the error norm is given by Eq. 40 for a given $c>\bar{c}$. So we can conclude that if $c>\bar{c}>0$, where $\bar{c}$ is defined in Eq. 39, bounded synchronization is achieved. $\square$

#### *Remark 3*

Note that for increasing values of the coupling strength *c*, the estimated error bound $\epsilon$ decreases (see Eq. 40 and Eq. 41), hence so does the relative synchronization error bound $\chi$ from Definition 2. As shown qualitatively earlier in Sect. 4 and numerically later in Sect. 7.3, this also implies that the coordination level of the network, captured by the group synchronization index $\rho _g$, increases.

**Fig. 9:**
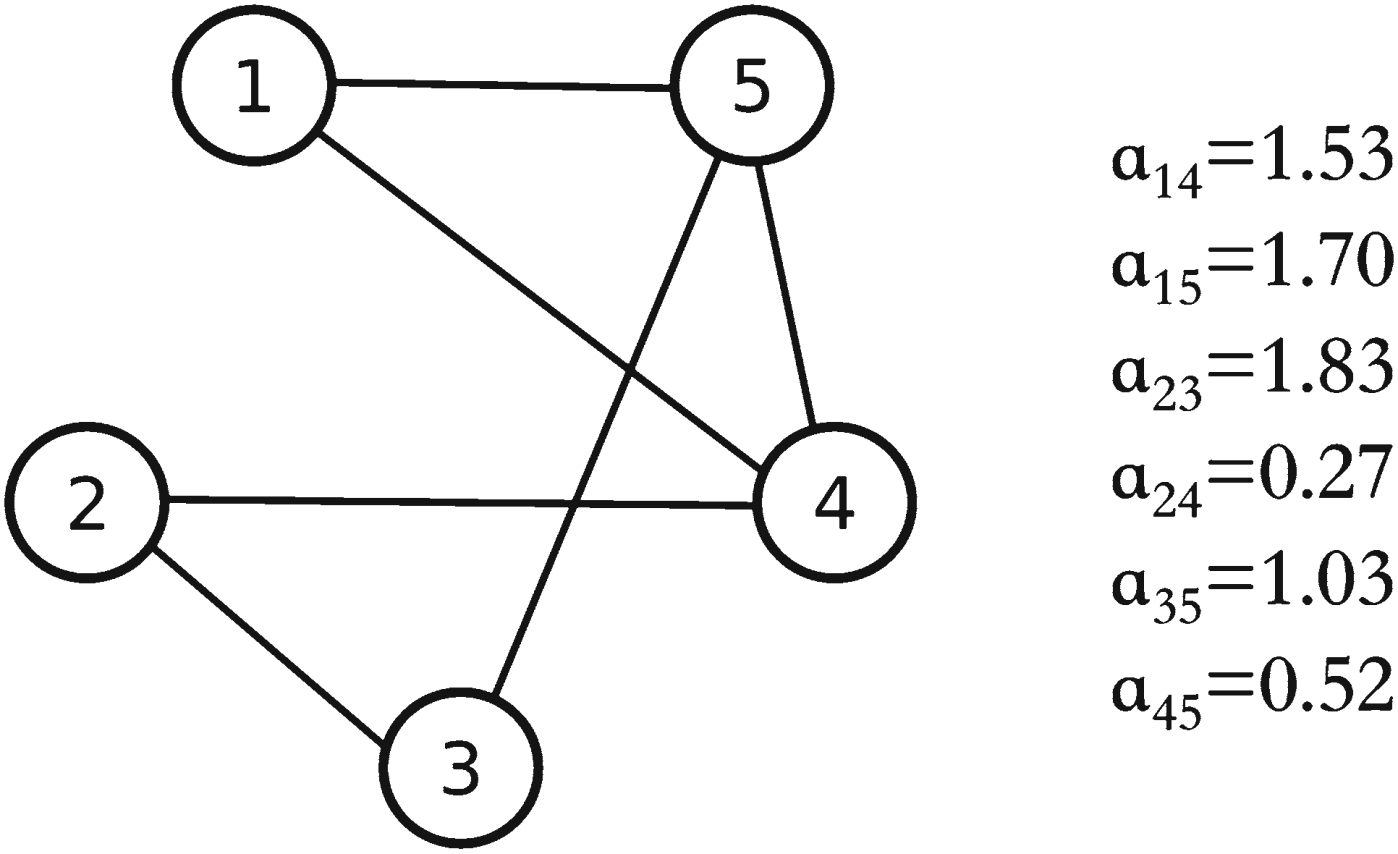
Underlying topology—simple connected weighted graph

### Numerical validation

As previously shown for a connected simple undirected network of *N* heterogeneous RvdP oscillators, by making use of contraction theory it is possible to guarantee bounded synchronization if we suppose that the underlying topology is an unweighted complete graph (all-to-all network). On the other hand, by making use of Lyapunov theory, bounded synchronization can be achieved regardless of the topology and the weights of the interconnections, although an assumption has to be made on one of the parameters of the nodes ($\gamma _i=\tilde{\gamma } \ \forall i \in [1,N]$).

In order to be able to study the most general case, we consider a weighted random graph of $N=5$ heterogeneous RvdP oscillators characterized by $\gamma _i=\tilde{\gamma } \ \forall i \in [1,N]$. The graph is generated by assuming an edge formation probability of 60%, and edge weights are randomly picked between 0 and 2 (Fig. 9). The parameters and the initial conditions of the nodes are selected as in Table 7. With this parameter choice, the variables needed to prove convergence according to Corollary 1 can be computed as $p_M=2.6$, $v_M=0.96$, $\bar{M}=7.6$, $\lambda _2 \left( L_{{\mathcal {N}}} \right) =0.4112$, $P_{11}=0.077$, $P_{22}=0.077$, $W_{11}=0.001$, $W_{22}=0.045$ and $\bar{c} = 1.4211$. Moreover, we set $T=500$ s.

**Fig. 10:**
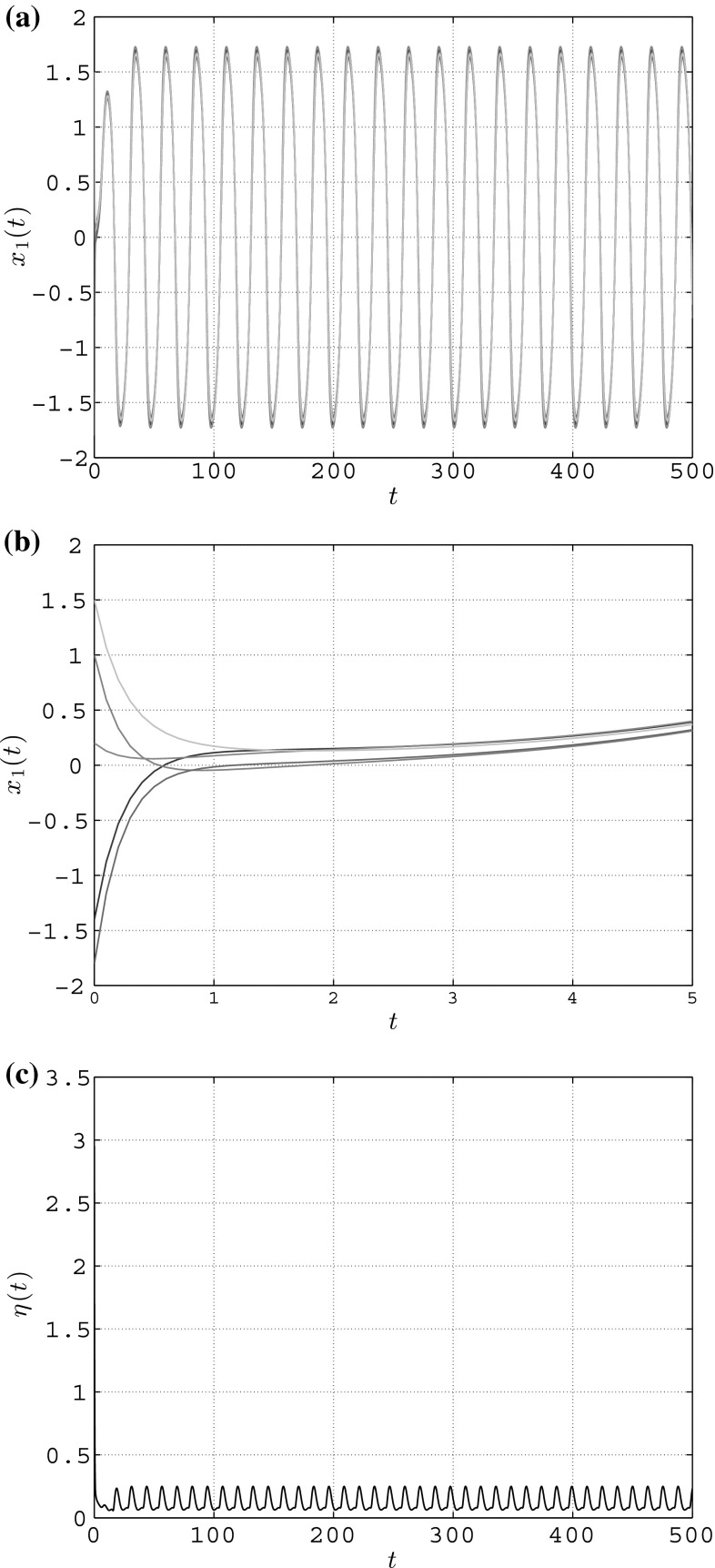
First state variable $x_{i_1}(t)$ and error norm $\eta (t)$ in a simple connected weighted network of $N=5$ heterogeneous RvdP oscillators, $\chi \simeq 0.065$—full state coupling ($c=1.45$). **a** First state variable—*blue line* node 1, *green line* node 2, *red line* node 3, *cyan line* node 4, *magenta line* node 5. **b** First state variable, zoom—*blue line* node 1, *green line* node 2, *red line* node 3, *cyan line* node 4, *magenta line* node 5. **c** Error norm

In Fig. 10, we show $x_1(t)$ for all the nodes in the network when they are connected through full state coupling with $c=1.45$. We also show the corresponding error norm $\eta (t)$. We observe that the nodes quickly converge toward each other with a residual error norm which is asymptotically bounded by $\epsilon \simeq 0.25$, as opposed to a maximum upper bound when the oscillators are uncoupled $\tilde{\eta } \simeq 3.89$ (corresponding to a relative synchronization error bound $\chi \simeq 0.065$).

**Table 7:** Numerical simulations—parameters and initial values for a network of $N=5$ heterogeneous RvdP oscillators

Node	$\alpha _i$	$\beta _i$	$\gamma _i$	$\omega _i$	$x_i(0)$
1	0.46	1.16	0.58	0.16	$[-1.4, +0.3]$
2	0.37	1.20	0.58	0.26	$[+1.0, +0.2]$
3	0.34	1.73	0.58	0.18	$[-1.8, -0.3]$
4	0.17	0.31	0.58	0.21	$[+0.2, -0.2]$
5	0.76	0.76	0.58	0.27	$[+1.5, +0.1]$

**Fig. 11:**
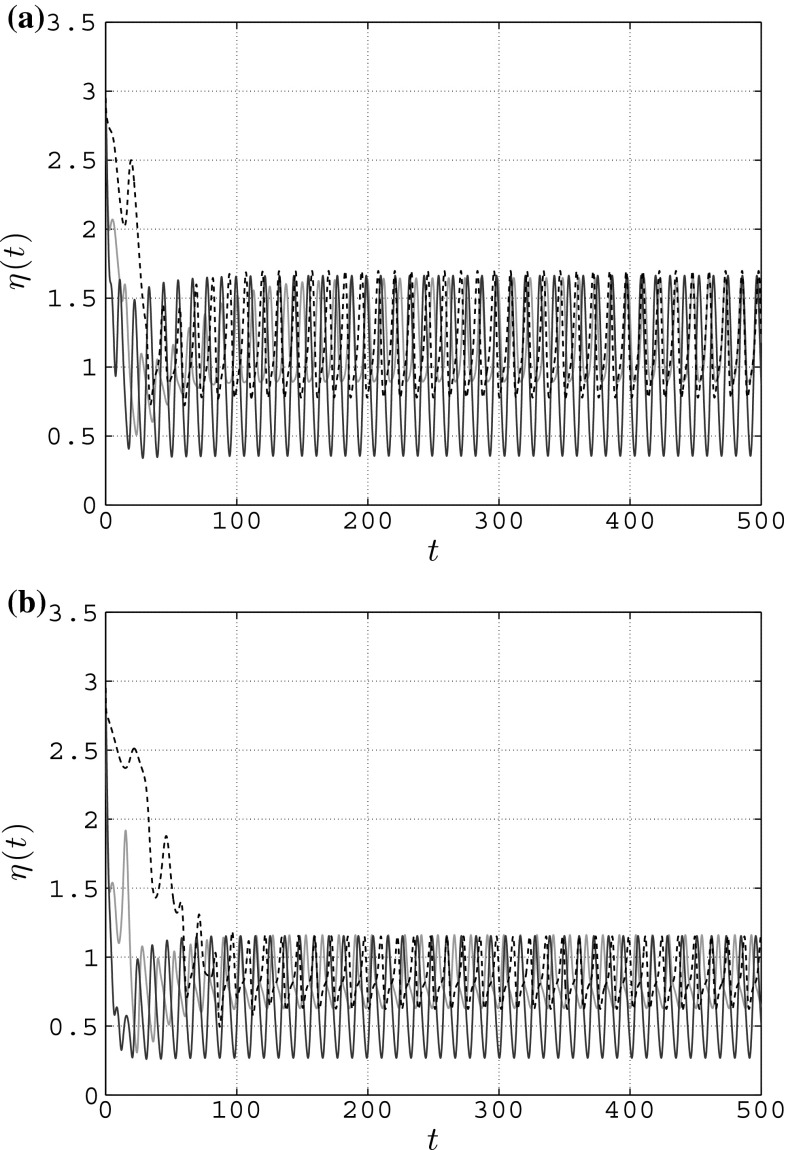
Error norm in a simple connected weighted network of $N=5$ heterogeneous RvdP oscillators—*magenta solid line* full state coupling (FSC), *blue solid line* partial state coupling (PSC), *black dashed line* HKB coupling (HKB). **a** Achievement of bounded synchronization with $\chi \simeq 0.44$—FSC: $c=0.085$, PSC: $c_1=c_2=0.13$, HKB: $a=b=-1, c=0.15$. **b** Achievement of bounded synchronization with $\chi \simeq 0.30$—FSC: $c=0.15$, PSC: $c_1=c_2=0.2$, HKB: $a=b=-1, c=0.3$

In Fig. 11a, we show that, when considering full state coupling, bounded synchronization can actually be achieved for smaller values of the coupling strength ($c=0.085$) and that it can also be achieved with the two other coupling functions presented earlier in this paper, yielding $\epsilon \simeq 1.7$ and $\chi \simeq 0.44$. Furthermore, in Fig. 11b we show how the upper bound $\epsilon$, and as a consequence, the relative synchronization error bound $\chi$ can be lowered at will by increasing the coupling strength. For instance, by setting $c=0.15$ in the full state coupling, the error norm bound reduces to $\epsilon \simeq 1.16$, corresponding to $\chi \simeq 0.30$.

**Fig. 12:**
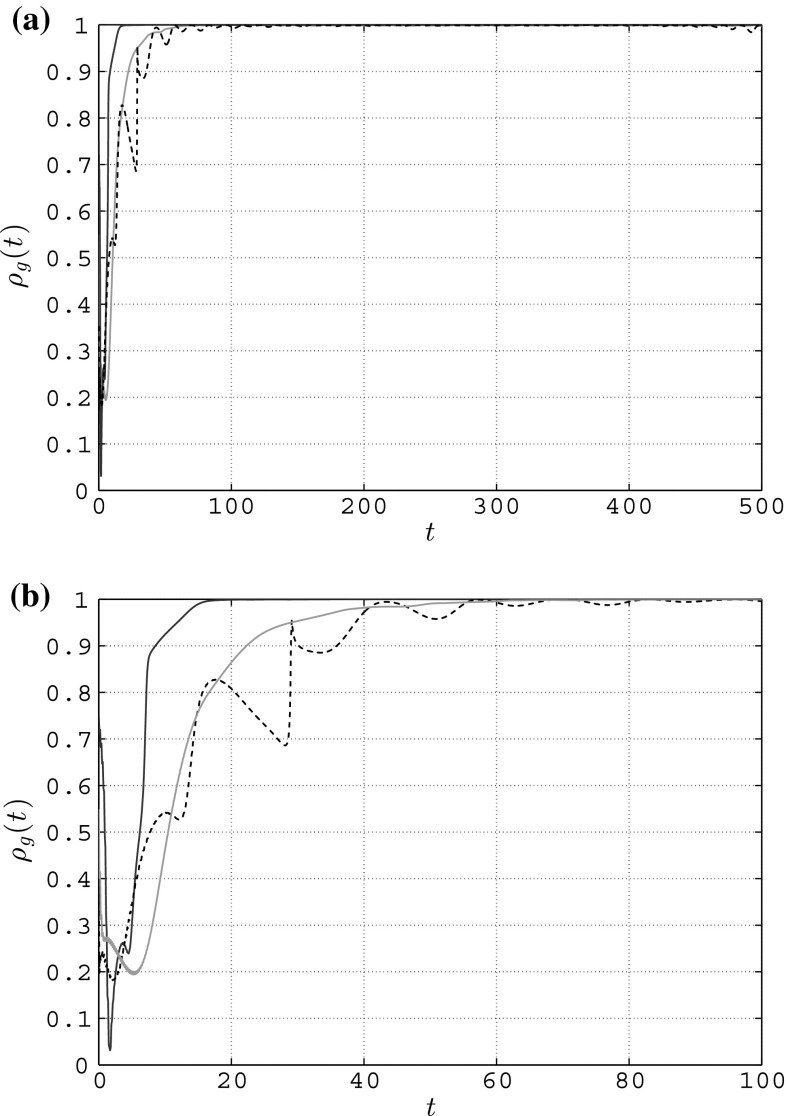
Group synchronization in a simple connected weighted network of $N=5$ heterogeneous RvdP oscillators—*magenta solid line* full state coupling ($c=0.085$), *blue solid line* partial state coupling ($c_1=c_2=0.13$), *black dashed line* HKB coupling ($a=b=-1, c=0.15$). **a** Group synchronization, **b** group synchronization—zoom

For the sake of completeness, in Fig. 12a we show the trend of the group synchronization obtained in each of three cases of coupling functions considered here: it is possible to appreciate how, after an initial transient, $\rho _g(t)$ reaches a much higher value, confirming what observed in Richardson et al. (2012). In particular, the trend obtained when considering an HKB coupling resembles the most that obtained in the real experiments involving human people. Indeed, from Fig. 12b it is possible to appreciate how, during the transient before synchronization reaches a high value, $\rho _g(t)$ exhibits oscillations as observed in the rocking chairs experiments only when using an HKB coupling, while its trend is mostly exponential with the other two couplings.

Finally, in Table 8 we show the link between the coupling strength *c*, the relative synchronization error bound $\chi$ as defined in Eq. 12, and the mean value of the group synchronization $\rho _g$ as defined in Eq. 19, when considering full state coupling (analogous results can be obtained when considering partial state coupling or HKB coupling). As expected (see Remark 3), increasing values of the coupling strength *c* correspond to decreasing values of the relative synchronization error bound $\chi$ and increasing values of the average of the group synchronization index $\rho _g$. This means that the stronger the interaction among all the nodes in the network, the smaller the distance among their trajectories, and the higher their coordination level.

**Table 8:** Link between coupling strength *c*, relative synchronization error bound $\chi$, and mean value of the group synchronization $\rho _g$ in a simple connected weighted network of $N=5$ heterogeneous RvdP oscillators connected through full state coupling

*c*	$\chi$	$\rho _g$
0.030	0.93	0.6394
0.040	0.81	0.7770
0.060	0.60	0.9056
0.070	0.51	0.9202
0.085	0.44	0.9236
0.150	0.30	0.9447
0.200	0.26	0.9791
0.300	0.19	0.9974
1.450	0.06	0.9999

Note that, for all the different values of *c*, the group synchronization index has been averaged over only the first 150 s of the simulation, despite its total duration being $T=500$ s. This has been necessary in order to appreciate the quantitative difference of the coordination level during the transient before $\rho _g(t)$ reaches a value almost equal to unity at steady-state

## Conclusion

We proposed a mathematical model based on a network of coupled heterogeneous RvdP oscillators to explain movement synchronization in a group of three or more people performing a joint oscillatory task. Each oscillator in the model is characterized by a different set of parameters to account for human-to-human variability. Three different coupling models, both linear and nonlinear, were investigated, and the effects of adding an external entrainment signal were analyzed. Also, we found analytical conditions for a connected simple undirected network to achieve bounded synchronization when considering full state coupling among the nodes, while we numerically verified that bounded synchronization can be achieved also when considering partial state coupling or HKB coupling. In particular, we observed that it is possible to replicate some of the synchronization features observed experimentally in the rocking chairs set-up described in Richardson et al. (2012) with all the three coupling functions proposed in this paper; the closest matching with the experimental data being achieved when connecting the nodes through a nonlinear HKB coupling function. The link between coupling strength, bounded synchronization, and coordination level of the network was also investigated, showing that, as expected from the theory of synchronization in complex networks, increasing the coupling reduces the synchronization error bound and improves group coordination.

Ongoing work is focused on exploiting the model analyzed in this paper to capture further experimental observations related to the coordination of hand movements in groups of more than two players. Also, research effort is being spent to obtain analytical conditions for synchronization of networks of RvdP oscillators with more general coupling functions.
